# Citrate Promotes Excessive Lipid Biosynthesis and Senescence in Tumor Cells for Tumor Therapy

**DOI:** 10.1002/advs.202101553

**Published:** 2021-11-07

**Authors:** Yangjing Zhao, Xia Liu, Fusheng Si, Lan Huang, Aiqin Gao, Wenli Lin, Daniel F. Hoft, Qixiang Shao, Guangyong Peng

**Affiliations:** ^1^ Department of Immunology Key Laboratory of Medical Science and Laboratory Medicine of Jiangsu Province School of Medicine Jiangsu University Zhenjiang 212013 P. R. China; ^2^ Division of Infectious Diseases Allergy & Immunology and Department of Internal Medicine Saint Louis University School of Medicine Saint Louis MO 63104 USA; ^3^ Department of Molecular Microbiology & Immunology Saint Louis University School of Medicine Saint Louis MO 63104 USA

**Keywords:** apoptosis, cellular senescence, chemotherapy, citrate, DNA damage response, lipid metabolism, MAPK, mTOR

## Abstract

Metabolic disorder is one of the hallmarks of cancers, and reprogramming of metabolism is becoming a novel strategy for cancer treatment. Citrate is a key metabolite and critical metabolic regulator linking glycolysis and lipid metabolism in cellular energy homeostasis. Here it is reported that citrate treatment (both sodium citrate and citric acid) significantly suppresses tumor cell proliferation and growth in various tumor types. Mechanistically, citrate promotes excessive lipid biosynthesis and induces disruption of lipid metabolism in tumor cells, resulting in tumor cell senescence and growth inhibition. Furthermore, ATM‐associated DNA damage response cooperates with MAPK and mTOR signaling pathways to control citrate‐induced tumor cell growth arrest and senescence. In vivo studies further demonstrate that citrate administration dramatically inhibits tumor growth and progression in a colon cancer xenograft model. Importantly, citrate administration combined with the conventional chemotherapy drugs exhibits synergistic antitumor effects in vivo in the colon cancer models. These results clearly indicate that citrate can reprogram lipid metabolism and cell fate in cancer cells, and targeting citrate can be a promising therapeutic strategy for tumor treatment.

## Graphical abstract

Reprogramming of metabolism is a novel strategy for cancer treatment. Extracellular citrate administration in tumor cells induces activation of the DNA damage response, MAPK, and mTOR signaling pathways. The activated cascades further induce excessive lipid biosynthesis and provoke senescence to impair tumor cell growth in vitro and in vivo. Targeting citrate can be a promising therapeutic strategy for tumor treatment.

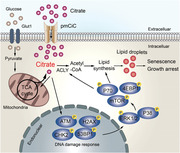

## 1 Introduction

Metabolic reprogramming in glucose and lipid metabolism is well‐recognized as a core cancer hallmark to fulfill increased energy demands for malignant transformation and progression.^[^ ^1^ ^]^ The reprogrammed glucose metabolism in cancer cells is characterized as exacerbated glucose uptake and aerobic glycolysis utilization leading to increased production of many metabolites and intermediates.^[^ ^2^ ^]^ Furthermore, cancer cells also show increased lipid synthesis, storage, and avidity achieved by de novo synthesis or exogenous uptake.^[^ ^3^ ^]^ The complex metabolic ecosystem in cancer cells not only satisfies energy generation for cell growth but also drives cancer therapeutic resistance.^[^ ^4^, ^5^ ^]^ Therefore, disrupting the vulnerable nutrient balance of tumor cells will provide therapeutic benefits and overcome drug resistance. In addition, dietary interventions as well as targeting key metabolic enzymes and intermediates have exerted potent tumor suppression, which is becoming a novel and promising strategy for cancer treatment.^[^ ^6^, ^7^, ^8^, ^9^ ^]^

Citrate is a critical metabolite, which is synthesized in mitochondria from acetyl‐CoA and oxaloacetate (OAA) by citrate synthase (CS) and enters the tricarboxylic acid (TCA) cycle. Besides the production of energy in TCA cycle, citrate can be transported into the cytoplasm through mitochondrial citrate carrier (mCiC) and catalyzed by ATP citrate lyase (ACLY) to generate acetyl‐CoA to provide the major source for lipid biosynthesis.^[^ ^10^, ^11^ ^]^ Furthermore, citrate is an important metabolic regulator involving various pathophysiological processes, including inflammation and cancer.^[^ ^12^, ^13^ ^]^ It is reported that intracellular citrate levels in cancer cells are reduced because of the low production of citrate in the mitochondria and increased cleavage by ACLY.^[^ ^14^ ^]^ Decreased levels of citrate in prostate cancer tissues and in blood of lung and pancreas cancer patients can potentially serve as an indicator of cancer aggressiveness and progression.^[^ ^15^, ^16^, ^17^ ^]^ In addition, the increasing intracellular concentration of citrate through exogenous sodium citrate (SCT) administration or ACLY inhibition has shown potent anti‐tumor activity on several cancer types.^[^ ^18^, ^19^ ^]^ Therefore, a better understanding of the precise effects and molecular mechanisms underlying citrate administration on tumor cells could provide an effective and alternative strategy for cancer treatment.

In this study, we analyzed the clinical relevance of ACLY with clinical outcomes in cancer patients. We further demonstrated that both SCT and citric acid (CA) treatments significantly inhibited tumor cell proliferation and growth in various tumor types. We then identified that citrate‐induced tumor suppression was due to the promotion of excessive lipid biosynthesis and induction of cell senescence in tumor cells. Furthermore, ATM‐associated DNA damage response (DDR) cooperated with MAPK and mTOR signaling pathways to control citrate‐induced tumor cell growth arrest and senescence. In addition, we demonstrated that citrate administration not only dramatically inhibited tumor growth and progression in vivo, but also exhibited synergistic antitumor effects with the combined conventional chemotherapy drugs in vivo in the colon cancer xenograft models. These findings suggest that citrate administration is an effective and promising therapeutic strategy against cancer.

## 2 Results

### 2.1 Increased ACLY Expression in Patients Predicts Poor Prognosis in Various Tumors

**Figure 1:**
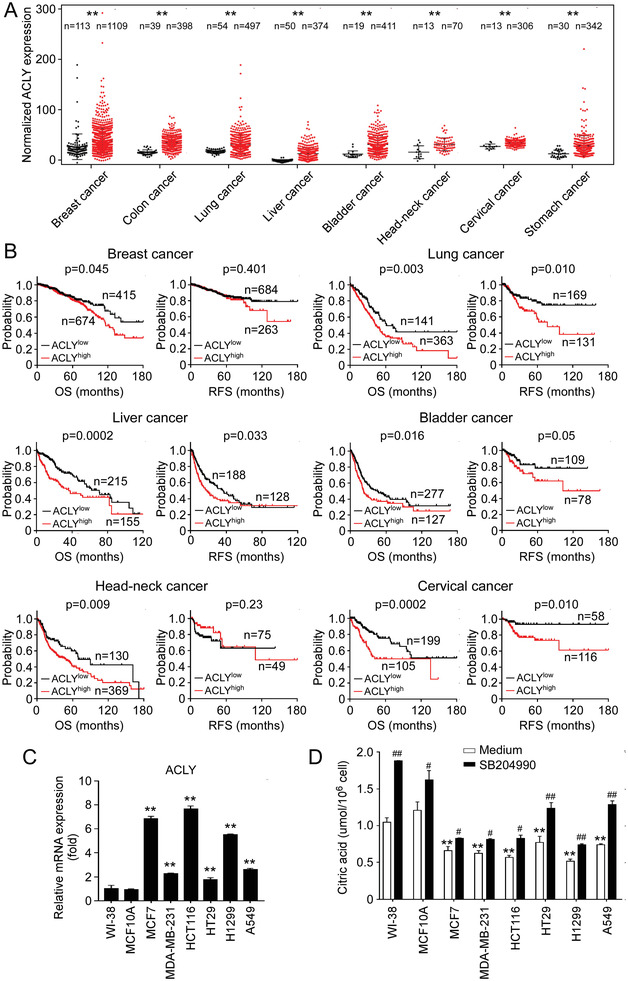
Increased ACLY expression in various tumor tissues is associated with poor clinical outcomes in cancer patients. A) ACLY mRNA expression was upregulated in tumor tissues compared with normal samples detected by RNA sequencing in eight tumor types. The sequencing data were obtained from the TCGA database. Black dots represent normal samples and red dots represent tumor samples. Data shown are mean ± SD. **p<0.01, between normal and tumor samples in each tumor type. B) OS and RFS analyses were performed in patients with low or high ACLY mRNA expression in six tumor types. The sequencing data were obtained from the TCGA database. The Kaplan–Meier plots were analyzed by the log‐rank test. C) High mRNA expression of ACLY in cancer cell lines compared with normal cell lines (WI‐38 and MCF10A). Total RNA was isolated from cells and gene expression was analyzed by real‐time qPCR. ACLY expression levels were normalized to *β*‐actin expression level and adjusted to the levels in WI‐38 cells (served as 1). **p<0.01, compared with WI‐38 cells. D) Inhibition of ACLY induced increases of intracellular CA in both human normal cell lines (WI‐38 and MCF10A) and multiple cancer cell lines. Citrate levels in indicated cells were detected by CA content assay kit after being treated with or without ACLY inhibitor SB204990 (10 µm) for 48 h. **p<0.01, compared with WI‐38 cells. ^#^ *p*<0.05 and ^##^ *p*<0.01, compared with the respective medium‐only group. Unpaired Student's *t*‐test was performed in (A). Log‐rank test was used to determine the statistical significance in (B). One‐way analysis of variance (ANOVA) was performed in (C) and (D).

ACLY functions as a citrate lyase and directly controls citrate levels in the cytoplasm.^[^ ^10^, ^11^ ^]^ Therefore, we investigated the expression levels of ACLY in various cancers and its correlations with the outcomes of cancer patients. We first obtained the gene expression and clinical data from The Cancer Genome Atlas (TCGA) database and then analyzed the ACLY expression in different types of cancers.^[^ ^20^ ^]^ We found that ACLY mRNA levels were obviously elevated in tumor tissues compared with that in respective normal tissues in all eight solid tumors, including breast invasive carcinoma, colon adenocarcinoma, lung cancer, hepatocellular carcinoma, bladder urothelial carcinoma, head and neck squamous cell carcinoma, cervical cancer and stomach adenocarcinoma (**Figure** **1**A). These data also strongly suggested a decreased level of citrate in tumor tissues. We next examined the associations between ACLY expression levels and patients’ survivals using the TCGA clinical data.^[^ ^20^ ^]^ The analyses revealed that the patients with relatively higher ACLY expression showing significantly poorer overall survival (OS) than those with lower ACLY expression in breast, lung, liver, bladder, head and neck, and cervical cancers (all *p*<0.05). In addition, higher ACLY expression levels in cancer patients were associated with shorter disease relapse free survivals (RFS) in lung, liver, bladder, and cervical cancers (Figure 1B). These results collectively suggest that increased ACLY in cancer patients is negatively correlated with clinical outcomes in cancers.^[^ ^21^, ^22^, ^23^ ^]^

To further dissect the potential relationship between ACLY expression and citrate level in tumor cells, we determined ACLY mRNA expression in different types of tumor cell lines, including breast cancer MCF7 and MDA‐MB‐231, colon cancer HCT116 and HT29, and lung cancer H1299 and A549 cell lines. We found high expression of ACLY in all the tumor cells compared with that in normal fibroblast WI‐38 cells and breast MCF10A cells (Figure 1C). In contrast, low levels of CA were detected in all the tumor cell lines compared with normal WI‐38 and MCF10A cells (Figure 1D). In addition, inhibition of ACLY activity by specific inhibitor SB204990 induced increased levels of intracellular CA in both tumor cells and normal control cells, suggesting internal regulation of citrate by ACLY in tumor cells (Figure 1D).

### 2.2 Citrate Inhibits Tumor Cell Proliferation and Growth Independent of Apoptosis

**Figure 2:**
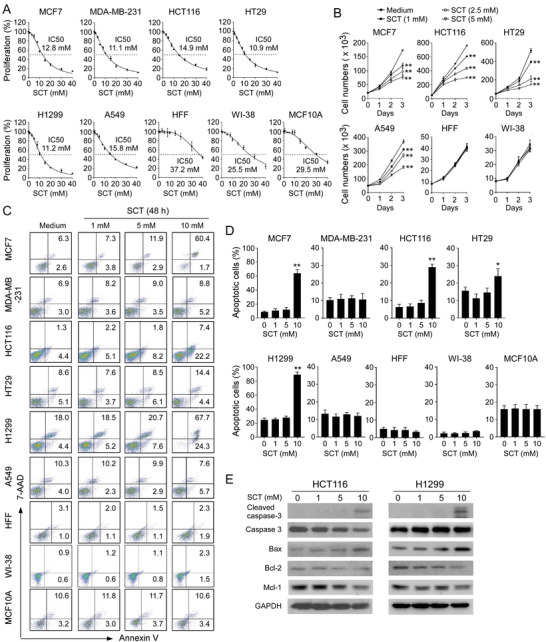
SCT inhibits tumor cell proliferation and growth. A) Multiple cancer cell lines were treated with increasing doses of SCT for 24 h and cell proliferation was measured by the MTT assay. The normal HFF, WI‐38, and MCF10A cells served as controls. The values of cell proliferation are shown as mean ± SD of six repeated wells. IC50 value for each cell type is a representative of three independent experiments. B) MCF7, HCT116, HT29, and A549 tumor cells and normal HFF, WI‐38, and MCF10A cells were seeded at optimized starting numbers in 24‐well plates in the presence of indicated concentrations of SCT. The cell growth was evaluated at different time points using cell number counting. Data shown are mean ± SD from three independent experiments. **p<0.01, compared with the medium‐only group. C,D) Low doses of SCT did not induce tumor cell apoptosis. Different types of tumor cells were treated with the indicated concentrations of SCT for 48 h. Normal HFF, WI‐38, and MCF10A cells were included as a control. Apoptotic cells were analyzed using the flow cytometric analysis after Annexin V and 7‐AAD double staining. Data shown (in D) are mean ± SD from three experiments with similar results. *p<0.05 and **p<0.01, compared with the medium‐only group. E) Only high dose of SCT induced apoptotic protein expression in tumor cells. HCT116 and H1299 cells were treated with/without the indicated concentrations of SCT for 24 h. Protein expressions of cleaved caspase‐3, caspase‐3, Bax, Bcl‐2, and Mcl‐1 were determined using the western blot analyses. ANOVA was performed in (B) and (D).

ACLY analysis results in cancer strongly indicate that citrate might be important for tumor growth and functions. The intracellular citrate mainly exists in three forms: divalent cation chelate, CA, and citrate anions (Cit3^−^).^[^ ^24^ ^]^ To test this possibility, we utilized SCT and CA to determine whether and how these two forms of citrates affect tumor cells. A panel of tumor cell lines were cultured in the presence of various concentrations of SCT or CA, and cell proliferation was measured by MTT and cell growth curve assays. We observed that both SCT and CA strongly inhibited tumor proliferation and growth in all the tumor cell lines, including breast cancer MCF7 and MDA‐MB‐231, colon cancer HCT116 and HT29, lung cancer H1299 and A549 cell lines, in a dose‐dependent manner (**Figure** **2**A,B, and Figure S1A,B, Supporting Information). Furthermore, sensitivities to citrate treatment were varied among different tumor cell lines for 24‐h culture, and the half maximal inhibitory concentration (IC50) values of SCT on tumor cell proliferation ranged from 10 to 15 mm, and the IC50 values of CA were 5 to 10 mm (Figure 2A and Figure S1A, Supporting Information). In contrast, both SCT and CA showed little or no effect on the cell proliferation and growth of human fibroblasts (HFF and WI‐38) and normal breast cell line (MCF10A) (Figure 2A,B, and Figure S1A,B, Supporting Information). In addition, suppression of tumor proliferation and growth in different types of tumor cell lines was further confirmed by the Edu proliferation assays (Figure S1C, Supporting Information).

To further identify the mechanism responsible for the suppression of tumor proliferation and growth induced by citrate, we determined whether SCT and CA can induce apoptosis in tumor cells.^[^ ^13^, ^25^, ^26^ ^]^ Different tumor cell lines were cultured in the various concentrations of SCT and CA for 24 and 48 h. We found that low concentrations of SCT (below 5 mm) or CA (4 mm) did not induce apoptotic cell populations in different types of tumor cells, although these concentrations of citrates strongly inhibited tumor growth and proliferation (Figure 2C,D, and Figure S2A,B, Supporting Information). However, treatment with 10 mm SCT significantly induced cell apoptosis in MCF7, HCT116, HT29, and H1299 tumor cells at both 24 and 48 h, suggesting that only high concentrations of citrate promote tumor apoptosis (Figure 2C,D, and Figure S2A, Supporting Information). To further confirm these results, we detected apoptosis‐associated protein expression in tumor cells after citrate treatments. We observed that only a high dose of SCT (10 mm) induced increases of cleaved caspase‐3 and Bax expression, as well as decreases of Bcl‐2/Bax ratio and Mcl‐1 in both HCT116 and HT1299 tumor cells (Figure 2E). These results indicate that suppressive effects on tumor cells mediated by low concentrations of citrate are independent of induction of apoptosis.

### 2.3 Citrate Induces Senescence and Initiates DNA Damage in Tumor Cells

**Figure 3:**
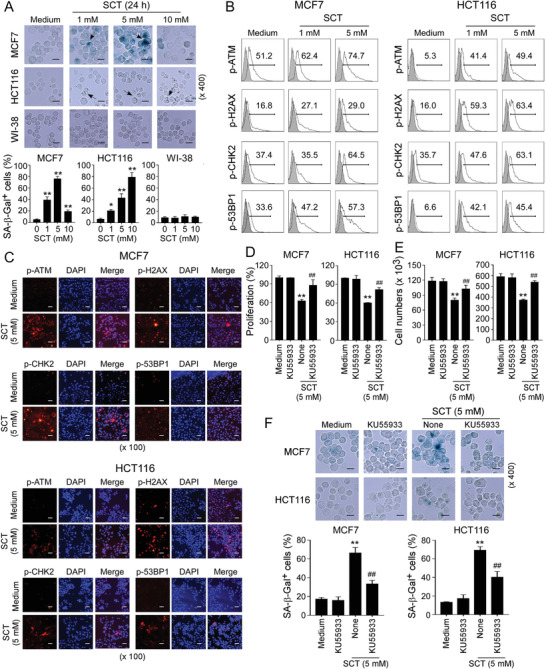
SCT induces DDRs and senescence in tumor cells. A) Extracellular SCT treatment increased senescent cell populations in tumor cells but not in normal cells. MCF7, HCT116, and WI‐38 cells were treated with the indicated concentrations of SCT for 24 h and then stained for SA‐*β*‐Gal. SA‐*β*‐Gal^+^ cells were shown with dark blue granules as indicated by the arrows. Data shown in histograms are mean ± SD from three independent experiments. *p<0.05 and **p<0.01, compared with the medium‐only group. Scale bar: 30 µm. B,C) Phosphorylated activations of key DNA damage molecules ATM, H2AX, 53BP1, and CHK2 in MCF7 and HCT116 tumor cells were detected by the flow cytometry analysis (in B) and the immunofluorescence staining (in C) after culture with indicated concentrations of SCT for 24 h. Scale bar: 100 µm. D,E) Inhibition of ATM signaling reversed citrate‐induced suppression on tumor cell proliferation and growth. MCF7 and HCT116 cells were pretreated with ATM inhibitor KU55933 (5 µm) for 24 h and then cultured with SCT (5 mm) for 48 h. Cell proliferation and growth were determined with the MTT assay (in D) and cell numbers counting (in E), respectively. Proliferation of tumor cells with medium only reversed as 100% (in C). Data shown are mean ± SD from three independent experiments. **p<0.01, compared with the medium‐only group. ^##^ *p*<0.01, compared with citrate treatment only group. (F) Blockage of ATM signaling prevented citrate‐induced tumor cell senescence. Cell treatment and procedure were identical to (D) and (E). Senescent cell populations were determined using the SA‐*β*‐Gal staining. Data shown in histograms are mean ± SD from three independent experiments. **p<0.01, compared with the medium‐only group. ^##^ *p*<0.01, compared with the citrate treatment group. Scale bar: 30 µm. ANOVA was performed in (A), (D), (E), and (F).

In addition to apoptosis, cellular senescence is another important cell fate characterized by stable proliferation arrest and activated DDR.^[^ ^27^, ^28^, ^29^ ^]^ We reasoned that citrate‐mediated suppression of tumor cell growth might be due to the promotion of tumor cell senescence. Senescence‐associated *β*‐galactosidase (SA‐*β*‐Gal) activity is a widely accepted biomarker for cellular senescence.^[^ ^28^, ^30^, ^31^, ^32^ ^]^ Both SCT and CA treatments significantly increased the percentages of SA‐*β*‐Gal^+^ cells in all cancer cells, indicating induction of tumor cell senescence. In contrast, even a high dose of SCT (10 mm) and CA (4 mm) did not induce SA‐*β*‐Gal expression in control, WI‐38, HFF, and MCF10A normal cells (**Figure** **3**A and Figure S3A,B, Supporting Information). In addition to the expression of SA‐*β*‐Gal, we found that SCT treatment dramatically promoted expression of cell cycle regulatory molecules P53, P21, and P16, as well as increased expression of genes encoding proinflammatory cytokines, including interleukin 1beta, 6, 8 (IL‐1*β*, IL‐6, IL‐8), and tumor necrosis factor alpha (TNF*α*), in tumor cells (Figure S4A,B, Supporting Information). Furthermore, SCT treatment markedly increased the accumulation of tumor cells in cell‐cycle arrest in G0/G1 phase (Figure S4C, Supporting Information).^[^ ^29^, ^33^, ^34^ ^]^ These results collectively suggest that citrate efficiently and selectively promotes senescence in tumor cells rather than in normal cells.

It has been established that the process of senescence is triggered and maintained by activated DDR pathways, including protein kinase ataxia telangiectasia mutated (ATM), histone H2AX, and their downstream mediators p53‐binding protein 1 (53BP1) and checkpoint kinase 2 (CHK2).^[^ ^35^ ^]^ We therefore investigated whether induction of DDR is involved in citrate‐induced tumor cell senescence. As expected, SCT treatment (5 mm) significantly induced phosphorylation and activation of ATM, H2AX, 53BP1, and CHK2 in MCF7 and HCT116 cells at 24 h using both flow cytometry analyses and immunofluorescence staining (Figure 3B,C). To further confirm ATM‐associated DDR participates in the citrate‐induced senescence processes in tumor cells, we investigated whether citrate‐induced senescence in tumor cells can be prevented by blocking DNA damage activation with the ATM‐specific pharmacological inhibitor KU55933. As expected, pretreatment of MCF7 and HCT116 cells with KU55933 significantly prevented the inhibition of tumor cell proliferation and growth induced by both SCT and CA treatments (Figure 3D,E, Figure S5A,B, Supporting Information). In addition, blockage of ATM activation with KU55933 also dramatically decreased the senescence induction in MCF7 and HCT116 cells induced by SCT and CA treatments (Figure 3F and Figure S5C, Supporting Information). Collectively, these results clearly indicate that initiation of DNA damage is critical and required for the citrate‐mediated tumor cell proliferation inhibition and senescence induction.

### 2.4 Citrate‐Induced Tumor Suppression and Senescence is Due to Excessive Lipid Biosynthesis in Tumor Cells

Citrate is a key intermediate in the TCA cycle and is theoretically assumed to exert negative feedback on glycolysis.^[^ ^12^, ^14^ ^]^ Therefore, we explored whether citrate‐mediated suppression and senescence induction in tumor cells is through the interference of cell glucose metabolism. We determined expression levels of key glucose metabolism‐associated enzymes in MCF7 and HCT116 cells treated by citrate using the quantitative real‐time PCR analysis. These genes include glucose transporter (Glut) 1 and Glut3, glycolysis‐related enzymes hexokinase 2 (HK2), glucose‐6‐phosphate isomerase (GPI), phosphofructokinase 1 (PFK1), triosephosphate isomerase 1 (TPI1), enolase 1 (ENO1), pyruvate kinase muscle 2 (PKM2), lactate dehydrogenase A (LDH*α*), and hypoxia inducible factor 1‐alpha (HIF1*α*).^[^ ^28^, ^32^ ^]^ Surprisingly, we did not observe any significant changes among these enzymes except a minor decrease of PFK1 in both MCF7 and HCT116 tumor cells treated by SCT (Figure S6A, Supporting Information). Furthermore, we also examined whether citrate treatment affects glucose uptake ability of tumor cells using the fluorescent glucose analog 2‐[N‐(7‐nitrobenz‐2‐oxa‐1,3‐diazol‐4‐yl) amino]‐2‐deoxy‐D‐glucose (2‐NBDG) labeling assay. Consistent with the above results, treatments with SCT and CA also did not markedly change glucose uptake of MCF7, HCT116, and HT29 cells (Figure S6B,C, Supporting Information). These results indicate that citrate treatment does not affect glucose metabolism in tumor cells.

**Figure 4:**
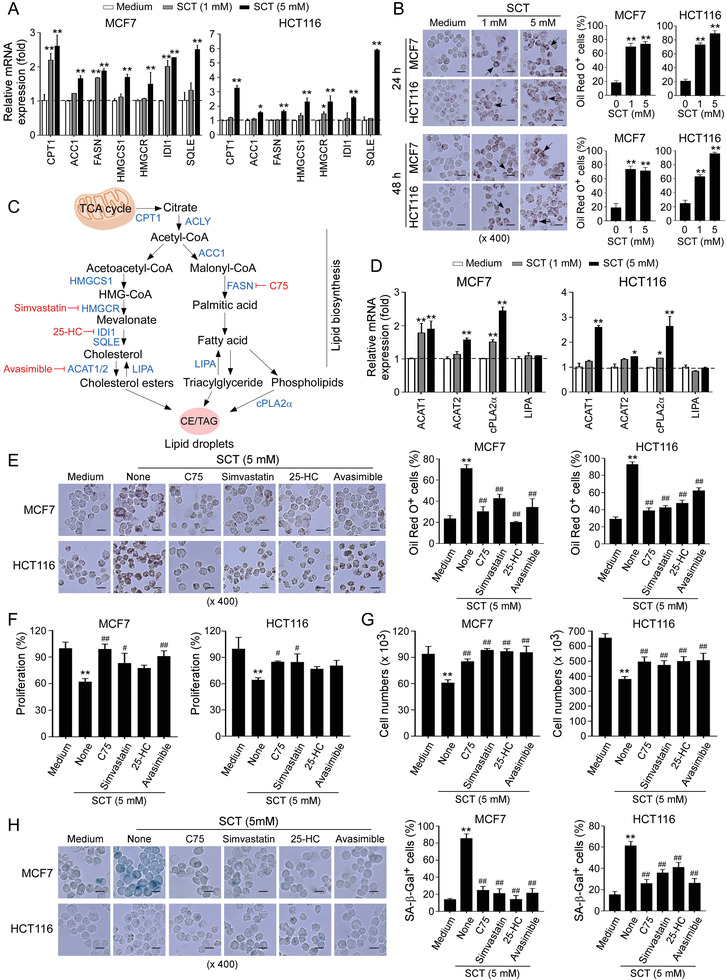
SCT‐induced excessive lipid biosynthesis is responsible for cell senescence and suppression in tumor cells. A) Increased gene expression levels of key enzymes in cholesterol synthesis (HMGCR, HMGCS1, SQLE, and IDI1), as well as fatty acid oxidation (CPT‐1) and synthesis (ACC1 and FASN) in tumor cells were induced with SCT treatment for 24 h. Total RNA was isolated from the treated tumor cells and gene expression was analyzed by real‐time qPCR. Expression levels of each gene were normalized to *β*‐actin expression level and adjusted to the levels in tumor cells treated with medium only (served as 1). Data shown are mean ± SD from three independent experiments with similar results. *p<0.05 and **p<0.01, compared with the medium‐only group. B) Accumulated LDs in tumor cells induced by SCT treatment. MCF7 and HCT116 cells were cultured in the presence of indicated concentrations of SCT for 24 or 48 h. The treated tumor cells were performed Oil Red O staining. The Oil Red O ^+^ tumor cells were identified with red granules as indicated by the arrows. Data shown in the right panels are means ± SD from three independent experiments. **p<0.01, compared with the medium‐only group. Scale bar: 30 µm. C) Schematic diagram of the lipid biosynthesis pathways. The key enzymes are shown in blue color and the specific pharmacological inhibitors used in this study are shown in red color. D) SCT treatment upregulated gene expression of key enzymes (ACAT1, ACAT2, and cPLA2*α*) involved in LD formation, but not hydrolase LIPA in tumor cells. MCF7 and HCT116 cells were treated with SCT (5 mm) for 24 h and mRNA expression levels of each gene were determined by the real‐time qPCR. The expression levels were normalized to *β*‐actin expression and adjusted to the levels in tumor cells with medium only. Data are mean ± SD from three independent experiments with similar results. *p<0.05 and **p<0.01, compared with the tumor cells in the respective medium only group. E) Blockage of the lipid synthesis reversed SCT‐induced LD accumulation in tumor cells. MCF7 and HCT116 cells were pretreated with the pharmaceutical inhibitors for lipid synthesis for 24 h, including C75 (5 µm), simvastatin (1 µm), 25‐HC (0.25 µg mL^−1^), or avasimible (1 µm), respectively. Tumor cells were then cultured in the presence of SCT (5 mm) for an additional 24 h and stained for Oil Red O. Data shown in the right panels are mean ± SD from three independent experiments with similar results. *p<0.05 and **p<0.01, compared with the medium‐only group. ^##^ *p*<0.01, compared with the citrate treatment only group. Scale bar: 30 µm. F,G) Inhibition of the lipid synthesis blocked citrate‐induced suppression on tumor cell proliferation and growth. Cell treatment and procedure were identical to (E). Cell proliferation and growth were determined with the MTT assay (in F) and cell numbers counting (in G), respectively. Proliferation of tumor cells with medium only served as 100% (in F). Data shown are mean ± SD from three independent experiments. **p<0.01, compared with the medium‐only group. ^#^ *p*<0.05 and ^##^ *p*<0.01, compared with the citrate treatment only group. H) Blockage of the lipid synthesis prevented citrate‐induced tumor cell senescence. Cell treatment and procedure were identical to (E). Senescent cell populations were determined using the SA‐*β*‐Gal staining. Data shown in histograms are mean ± SD from three independent experiments. **p<0.01, compared with the medium‐only group. ^##^ *p*<0.01, compared with the citrate treatment only group. Scale bar: 30 µm. ANOVA was performed in (A–H).

Citrate can be metabolized in the cytoplasm by ACLY to generate acetyl‐CoA for de novo lipogenesis, which is catalyzed by activated acetyl‐CoA carboxylase (ACC), the first enzyme for fatty acid synthesis.^[^ ^36^ ^]^ We next determined whether citrate treatment affects lipid metabolism in tumor cells. We analyzed a panel of key enzymes related to cholesterol and fatty acid synthesis and catabolism in tumor cells, including carnitine palmitoyltransferase I (CPT1), ACC1, fatty acid synthase (FASN), 3‐hydroxy‐3‐methylglutaryl‐CoA synthase 1 (HMGCS1), 3‐hydroxy‐3‐methyl‐glutaryl‐CoA reductase (HMGCR), isopentenyl‐diphosphate delta isomerase 1 (IDI1), and squalene monooxygenase (SQLE).^[^ ^32^ ^]^ We found that treatments with both SCT and CA significantly promoted the expression of these lipid metabolism‐associated enzymes in MCF7 and HCT116 tumor cells (**Figure** **4**A and Figure S7A, Supporting Information). Lipid droplets (LD) are intracellular storage organelles of neutral lipids, including cholesteryl ester (CE) and triglyceride (TAG), which can be detected by a fat‐soluble diazol dye Oil Red O staining.^[^ ^37^, ^38^ ^]^ We found that treatments with SCT and CA increased LD accumulation in different types of tumor cells, including MCF7 and HCT116 tumor cells, but not in control normal HFF, WI‐38, and MCF10A cells (Figure 4B and Figure S7B–E, Supporting Information). We also utilized the lipophilic fluorescent dye Bodipy 493/503 to evaluate the amounts of lipids in senescent tumor cells treated by citrate.^[^ ^39^ ^]^ Consistent with the Oil Red O staining results, SCT‐induced senescent MCF7 and HCT116 tumor cells displayed more fluorescence intensity than that of tumor cells cultured in medium only (Figure S7F, Supporting Information). To further identify the accumulation of lipid in tumor cells induced by citrate, we determined total cholesterol and free fatty acid in senescent tumor cells. We found significant increases in total cholesterol and free fatty acid in different types of tumor cells treated by SCT (Figure S7G,H, Supporting Information). These results clearly suggest that citrate induces high levels of lipid metabolism in tumor cells.

To further dissect the molecular processes responsible for the accumulated LDs in tumor cells induced by citrate, we detected the key enzymes involved in both synthesis and degradation of CE, TAG, and phospholipids, including ACAT acetyl‐CoA acetyltransferase 1 and 2 (ACAT1 and ACAT2) that convert cholesterol to CE, cytosolic phospholipase A2*α* (cPLA2*α*) that catalyzes the hydrolysis of phospholipids for LDs formation, and lipase A (LIPA) that hydrolyzes neutral lipids (Figure 4C).^[^ ^36^, ^40^, ^41^ ^]^ We observed that citrate treatment upregulated expression of ACAT1, ACAT2, and cPLA2*α* in citrate‐treated MCF7 and HCT116 cells. However, citrate did not change the expression of hydrolase LIPA in treated tumor cells (Figure 4D and Figure S8A, Supporting Information). To confirm the functional importance of increased lipid metabolism in citrate‐induced inhibitory capacity in tumor cells, we utilized the loss‐of‐function strategy with the specific pharmacological inhibitors targeting cholesterol and fatty acid metabolic pathways and then determined whether blockage of lipid metabolism can prevent citrate‐induced tumor cell senescence and suppression (Figure 4C). We found that pretreatment of tumor cells with general lipid biosynthesis inhibitors (C75, simvastatin, 25‐HC, and avasimible) markedly decreased LD accumulation in MCF7 and HCT116 tumor cells induced by citrate (Figure 4E and Figure S8B,C, Supporting Information). The optimized concentrations of those pharmacological inhibitors neither affected tumor cell viability and proliferation nor induced cell senescence (Figure S8D–F, Supporting Information). However, pretreatment with these lipid biosynthesis inhibitors could not only reverse proliferation and growth inhibition but also prevent senescence induction in tumor cells induced by SCT and CA (Figure 4F–H and Figure S8G–I, Supporting Information). Collectively, our results indicate that excessive lipid biosynthesis is responsible and required for citrate‐induced tumor cell growth suppression and senescence induction.

### 2.5 Citrate Treatment Induces Selective Modulation of MAPK and mTOR Signaling Pathways in Tumor Cells

**Figure 5:**
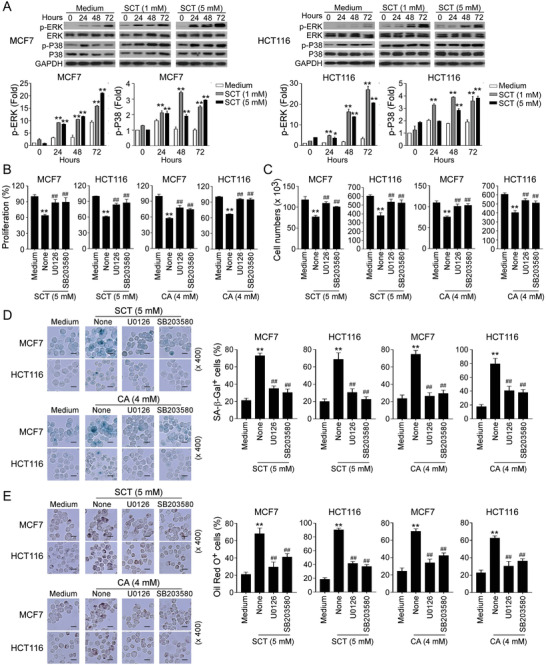
ERK and p38 MAPK signaling pathways control the molecular process of citrate‐induced tumor cell senescence. A) SCT treatment significantly induced phosphorylation of ERK1/2 and P38 in tumor cells. MCF7 and HCT116 cells were cultured for indicated time points with or without SCT (1 or 5 mm), and cell lysates were prepared for western blot analyses. Phosphorylated ERK1/2 and P38 protein levels shown in the histograms were quantitatively analyzed and compared against the GAPDH expression levels with a densitometer. Results shown are mean ± SD from three independent experiments. *p<0.05 and **p<0.01, compared with the medium‐only group. B,C) Pretreatment of tumor cells with ERK1/2 and P38 inhibitors U0126 and SB203580 significantly prevented citrate‐induced suppression of tumor cell proliferation and growth. MCF7 and HCT116 cells were pretreated with or without U0126 (4 µm) or SB203580 (5 µm) for 24 h and then further cultured in the presence of SCT or CA for 48 h. Cell proliferation and growth were determined using the MTT (in B) and cell numbers counting (in C) assays, respectively. Data shown are mean ± SD from three independent experiments. **p<0.01, compared with the medium‐only group. ^##^ *p*<0.01, compared with the citrate treatment only group. D) Blockage of the ERK1/2 and P38 signaling prevented citrate‐induced tumor cell senescence. MCF7 and HCT116 cells were pretreated with or without U0126 (4 µm) or SB203580 (5 µm) for 24 h and further cultured in the presence of SCT or CA for 24 h and then stained for SA‐*β*‐Gal. Data shown in the right panels are mean ± SD from three independent experiments. **p<0.01, compared with the medium‐only group. ^##^ *p*<0.01, compared with the citrate treatment only group. Scale bar: 30 µm. E) Blockage of the ERK1/2 and P38 prevented citrate‐induced LD accumulation in tumor cells. Cell treatment and procedure were identical to (D). Oil Red O staining was performed to detect LD accumulation. Data shown in the histograms are mean ± SD from three independent experiments. **p<0.01, compared with the medium‐only group. ^##^ *p*<0.01, compared with the citrate treatment only group. Scale bar: 30 µm. ANOVA was performed in (A–E).

Our previous studies also demonstrated that ERK1/2 and p38 MAPK signaling control the molecular process of human regulatory T cell (Treg) and tumor cell‐induced senescence in responder T cells.^[^ ^29^, ^31^, ^33^, ^39^, ^42^ ^]^ Therefore, we reasoned that MAPK signaling may also be involved in citrate‐induced tumor cell senescence. As expected, we found that SCT treatment significantly enhanced phosphorylation and activation of ERK1/2 and p38 in both MCF7 and HCT116 tumor cells at different time points (**Figure** **5**A). To further determine the regulatory role of ERK1/2 and p38 signaling in controlling citrate‐induced suppression and senescence in tumor cells, we utilized the specific pharmacological inhibitors U0126 (ERK1/2 inhibitor) and SB203580 (p38 inhibitor) to block MAPK signaling in tumor cells. We found that blockage of MAPK signaling with the inhibitors U0126 and SB203580 significantly reversed the suppression of cell proliferation and growth in MCF7 and HCT116 tumor cells induced by SCT and CA (Figure 5B,C). Furthermore, pretreatment of tumor cells with U0126 or SB203580 markedly prevented the induction of senescence in MCF7 and HCT116 tumor cells induced by both SCT and CA (Figure 5D). Given that LD accumulation is responsible for citrate‐induced tumor cell senescence, we next determined whether MAPK signaling is also involved in the lipid metabolism of tumor cells. We observed that blockage of ERK1/2 and p38 MAPK signaling pathways markedly reduced LD accumulation in MCF7 and HCT116 cells induced by SCT and CA (Figure 5E). These results suggest that activation of ERK1/2 and p38 MAPK pathways controls the molecular process of citrate‐induced growth suppression and senescence in tumor cells.

**Figure 6:**
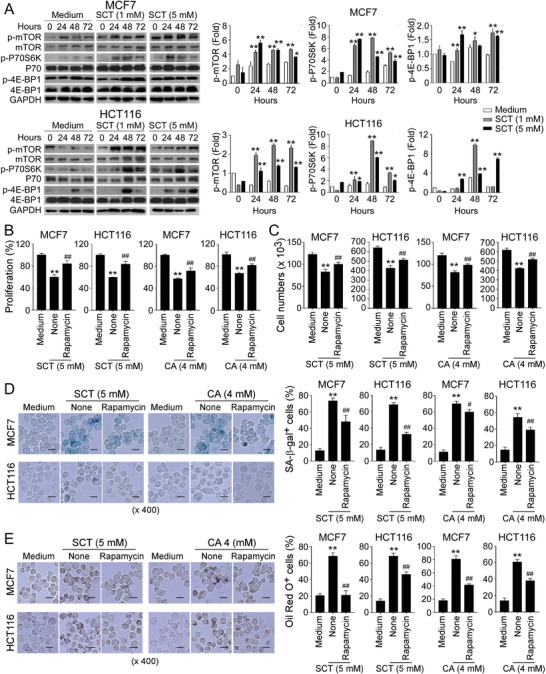
mTOR signaling involves tumor cell senescence induced by citrate treatment. A) Phosphorylated activation of mTOR signaling pathway in tumor cells was induced by SCT treatment. Treatment and procedure of MCF7 and HCT116 cells were identical to (Figure 5A). Phosphorylated mTOR, p70, and 4E‐BP1 protein levels shown in the right histograms were quantitatively analyzed and compared against the GAPDH expression levels with a densitometer. Results shown are mean ± SD from three independent experiments. *p<0.05 and **p<0.01, compared with the medium‐only group. B,C) Inhibition of mTOR signaling by pharmacological inhibitor rapamycin markedly reversed citrate‐induced inhibition of tumor cell proliferation and growth. MCF7 and HCT116 cells were pretreated with rapamycin (50 nm) for 24 h, and then cultured in the presence of SCT (5 mm) or CA (4 mm) for an additional 48 h. Cell proliferation and growth were determined by the MTT assay (in B) and cell numbers counting (in C), respectively. Data shown are mean ± SD from three independent experiments. **p<0.01, compared with the medium‐only group. ^##^ *p*<0.01, compared with the citrate treatment only group. D) Pretreatment of tumor cells with rapamycin significantly prevented citrate‐induced tumor cell senescence. MCF7 and HCT116 cells were pretreated with or without rapamycin (50 nm) for 24 h and then further cultured in the presence of SCT or CA for 24 h. Senescent cell populations were determined using the SA‐*β*‐Gal staining. Data shown in the histograms are mean ± SD from three independent experiments. **p<0.01, compared with the medium‐only group. ^#^ *p*<0.05 and ^##^ *p*<0.01, compared with the citrate treatment only group. Scale bar: 30 µm. E) Blockage of mTOR activation markedly prevented citrate‐induced LD accumulation in tumor cells. Cell treatment and procedure were identical to (D). Oil Red O staining was performed to detect LD accumulation. Data shown in the histograms are mean ± SD from three independent experiments. **p<0.01, compared with the medium‐only group. ^##^ *p*<0.01, compared with the citrate treatment only group. Scale bar: 30 µm. ANOVA was performed in (A–E).

In addition to MAPK signaling, mTOR kinase signaling is another signaling pathway that regulates cell metabolism and senescence.^[^ ^32^, ^43^ ^]^ We next investigated whether mTOR signaling is also involved in citrate‐induced tumor inhibition. We found that SCT treatment significantly induced the phosphorylation of mTOR, and its downstream substrates p70S6K and 4E‐BP1 in MCF7 and HCT116 cells, indicating activation of mTOR pathway in tumor cells after citrate treatment (**Figure** **6**A). Furthermore, blockage of mTOR signaling in tumor cells with the inhibitor rapamycin dramatically reversed the inhibition on cell proliferation and growth and prevented senescence induction in MCF7 and HCT116 tumor cells induced by SCT and CA (Figure 6B–D). In addition, pretreatment of tumor cells with rapamycin markedly decreased LD accumulation in MCF7 and HCT116 cells induced by both SAT and CA (Figure 6E). These results strongly suggest that both MAPK and mTOR signaling pathways are important and involved in citrate‐induced cell senescence and growth inhibition in tumor cells.

### 2.6 ATM‐Associated DNA Damage Response Cooperates with MAPK and mTOR Signing Pathways in Citrate‐Induced Tumor Cell Senescence and Inhibition

**Figure 7:**
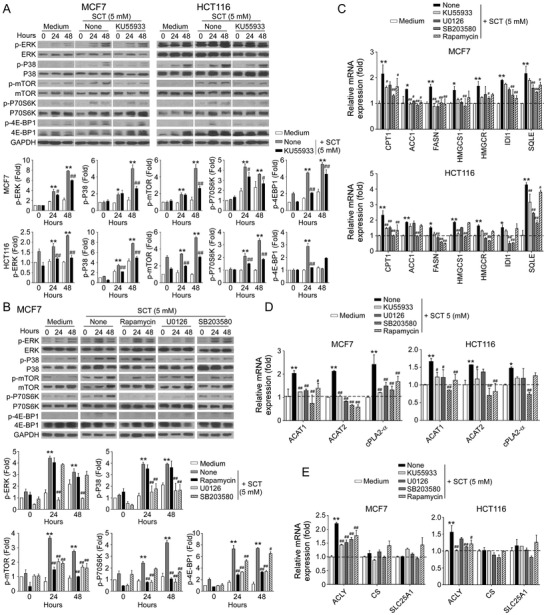
The causative regulations between DDR, MAPK, and mTOR signaling pathways for citrated‐induced excessive lipid metabolism and senescence in tumor cells. A) Treatment with ATM inhibitor KU55933 significantly prevented citrate‐induced ERK1/2, P38, mTOR, p70, and 4E‐BP1 phosphorylation in tumor cells using western blot analyses. MCF7 and HCT116 cells were pretreated with or without KU55933 (5 µm) for 24 h, and then cultured in the presence of SCT (5 mm) for 24 or 48 h. Protein levels of phosphorylated ERK1/2, P38, mTOR, p70, and 4EBP1 were quantitatively analyzed and compared against the GAPDH expression levels with a densitometer. Results shown in the histograms are mean ± SD from three independent experiments. *p<0.05 and **p<0.01, compared with the medium‐only group. ^#^ *p*<0.05 and ^##^ *p*<0.01, compared with the citrate treatment only group. B) Inhibition of ERK1/2 or P38 signaling pathways by specific pharmacological inhibitors markedly blocked citrate‐induced mTOR signaling activation in tumor cells. MCF7 and HCT116 cells were pretreated with inhibitors rapamycin (50 nm), U0126 (4 µm), or SB203580 (5 µm) for 24 h, and then treated with SCT (5 mm) for different time points. Phosphorylation of ERK1/2, P38 mTOR, p70, and 4EBP1 was determined by western blot analyses. Results shown in the histograms are mean ± SD from three independent experiments. **p<0.01, compared with the medium‐only group. ^#^ *p*<0.05 and ^##^ *p*<0.01, compared with the citrate treatment only group. C) Inhibition of MAPK or mTOR signaling by specific inhibitors significantly suppressed the increased gene expression levels of key enzymes in cholesterol and fatty acid synthesis (HMGCR, HMGCS1, SQLE, and IDI1, CPT‐1, ACC1, and FASN) in tumor cells induced by SCT treatment. Cell treatment and procedure were identical to (A) and (B). Gene expression in treated tumor cells was analyzed by real‐time qPCR. Expression levels of each gene were normalized to *β*‐actin expression level and adjusted to the levels in tumor cells treated with medium only (served as 1). Data shown are mean ± SD from three independent experiments with similar results. *p<0.05 and **p<0.01, compared with the medium‐only group. ^#^ *p*<0.05 and ^##^ *p*<0.01, compared with the citrate treatment only group. D,E) Blockage of the MAPK or mTOR signaling reversed the SCT‐induced upregulation of key enzymes (ACAT1, ACAT2, and cPLA2*α*) involved in LD formation (in D), and enzymes in citrate hydrolysis (in E) in tumor cells. Cell treatment, procedure, and data analysis were identical to (C). Data shown are mean ± SD from three independent experiments with similar results. *p<0.05 and **p<0.01, compared with the medium only group. ^#^ *p*<0.05 and ^##^ *p*<0.01, compared with the citrate treatment only group. ANOVA was performed in (A–E).

We have shown that initiation of ATM‐associated DDR is the cause for tumor cell senescence induced by citrate (Figure 3). We next explored the potential causative relationships and interactions among ATM‐associated DNA damage, MAPK and mTOR signaling pathways in the process of tumor cell senescence mediated by citrate. We first determined how blockage of ATM‐associated DNA damage affects MAPK, and mTOR signaling pathways during citrate‐mediated tumor cell senescence. Blocking ATM activation with KU55933 markedly prevented the phosphorylation of ERK1/2 and p38, as well as mTOR, p70S6K, and 4E‐BP1 in senescent MCF7 and HCT116 tumor cells induced by citrate, suggesting that ATM signaling is upstream of MAPK and mTOR signaling pathways during citrate‐induced tumor cell senescence (**Figure** **7**A). We next explored the reciprocal interactions between MAPK and mTOR signaling pathways in citrate‐induced senescent tumor cells. We observed that treatment with U0126 and SB203580 to block MAPK signaling significantly decreased the phosphorylation of mTOR, p70S6K, and 4EBP1 in senescent MCF7 cells induced by SCT treatment. However, blockage of mTOR signaling with rapamycin did not influence the phosphorylation and activation of ERK1/2 or p38 (Figure 7B). These studies collectively indicate the causative regulations and cross‐talks between ATM‐associated DNA damage initiation, MAPK, and mTOR signaling activation during the citrate‐induced tumor cell senescence.

We next determined whether these signaling pathways also control citrate‐mediated over‐activation of lipid metabolism in tumor cells. We found that pretreatment of MCF7 and HCT116 tumor cells with KU55933, U0126, SB203580 or rapamycin significantly reversed the citrate‐induced increase in the expression of enzymes involved in fatty acid and cholesterol synthesis and catabolism in tumor cells (Figure 7C). Furthermore, blockage of ATM, MAPK, and mTOR signaling pathways with inhibitors also prevented citrate‐induced overexpression of ACAT1/2 and cPLA2*α* for LD formation in MCF7 and HCT116 tumor cells (Figure 7D). In addition, SCT treatment could upregulate ACLY gene expression but did not affect citrate synthesis (CS) or mitochondrial citrate carrier SLC25A1 expression in MCF7 and HCT116 tumor cells. Moreover, blockages of ATM, MAPK, and mTOR signaling with respective inhibitors prevented citrate‐induced upregulation of ACLY in tumor cells (Figure 7E). These results indicated that ATM‐associated DDR, MAPK, and mTOR signaling pathways control the promoted lipid synthesis and senescence induction in tumor cells mediated by citrate.

### 2.7 Citrate Inhibits Tumorigenesis of Colon Cancer In Vivo

**Figure 8:**
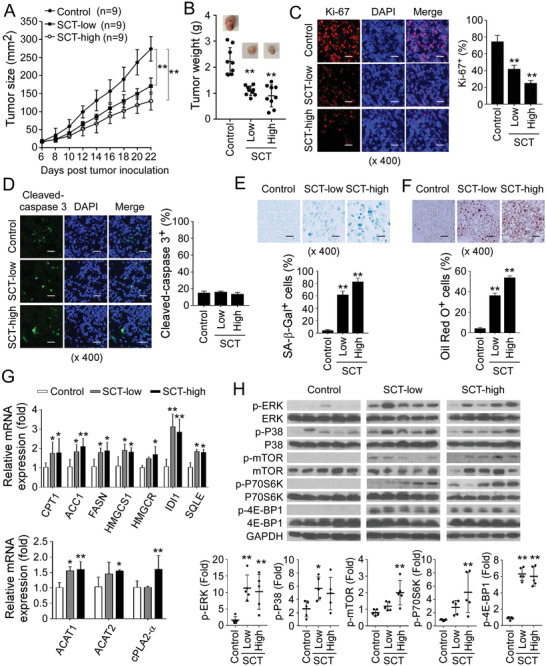
SCT inhibits tumor growth and progression in vivo in a colon cancer xenograft model. A,B) SCT treatment dramatically inhibited tumor growth in Rag1^−/−^‐immunodeficient mice. HCT116 cells (4.2 × 10^6^/mouse) were subcutaneously injected into Rag1^−/−^ mice. After 6 days of tumor injection, solvent control or SCT (15 mg kg^−1^ or 30 mg kg^−1^ body weight) were given by intraperitoneal injection at every other day for 16 days. Tumor volumes were measured and presented as mean ± SD (in A)(n = 9 mice/group). Representative images of the xenograft tumors obtained from the indicated groups at the endpoint of the experiments (day 22) are shown (in B). In addition, results shown are mean ± SD of the tumor weights from the indicated groups at the endpoint of the experiments (n = 9 mice/group). **p<0.01, compared with the solvent control injection group. C) SCT treatment significantly decreased Ki‐67^+^ cell populations in the tumor tissues using an immunofluorescence assay. Left panels are representative images of Ki‐67 expression in tumor tissues from different groups. Right panel is the summary of mean ± SD of Ki‐67^+^ cell fractions per high microscope field (× 400) in the tumor tissues from 9 mice of each group. **p<0.01, compared with the PBS control treatment mice. Scale bar: 30 µm. D) SCT treatment did not induce cleaved caspase‐3^+^ cell populations in tumor tissues using the immunofluorescence assay. Left panels are representative images of cleaved caspase‐3 expression in frozen tumor tissues from different groups. Right panel is the summary of mean ± SD of cleaved caspase‐3^+^ cell fractions per high microscope field (× 400) in the tumor tissues from 9 mice of each group. Scale bar: 30 µm. E) Large amounts of senescent tumor cells were observed in tumor tissues in SCT‐treated mice. SA‐*β*‐Gal expression was determined in the tumor frozen tissues from different groups at the endpoint of the experiment. Data shown in the histograms are mean ± SD of SA‐*β*‐Gal^+^ cell numbers per high microscope field (× 400) in the tumor tissues from 9 mice of each group. **p<0.01, compared with the control group. Scale bar: 30 µm. F) SCT treatment dramatically increased LD accumulation in tumor tissues from tumor‐bearing mice. Data shown in the lower histograms are mean ± SD of Oil Red O^+^ cell numbers per high microscope field (× 400) in the tumor tissues from 9 mice of each group. **p<0.01, compared with the control group. Scale bar: 30 µm. G) SCT administration increased gene expression of key enzymes involved in cholesterol and fatty acid synthesis (HMGCR, HMGCS1, SQLE, and IDI1, CPT‐1, ACC1, and FASN) and LD formation (ACAT1, ACAT2, and cPLA2*α*) in tumor tissues using the real‐time qPCR analyses. Expression levels of each gene were normalized to *β*‐actin expression levels and adjusted to the levels in the control group (served as 1). Data shown are mean ± SD from the tumor tissues from 9 mice of each group. *p<0.05 and **p<0.01, compared with the control group. H) SCT administration induced the phosphorylation of ERK1/2 and P38 in tumor tissues using western blot analyses. Phosphorylated ERK1/2 and P38 protein levels shown in the lower scatter diagrams were quantitatively analyzed and compared against the GAPDH expression levels with a densitometer. Every single dot represents one sample from an individual mouse. Results shown in the scatter diagrams are mean ± SD from three independent experiments. *p<0.05 and **p<0.01, compared with the control group. ANOVA was performed in (A–H).

Our in vitro studies have clearly shown that citrate can inhibit tumor proliferation and growth. We next performed in vivo studies to determine whether administration of citrate can inhibit tumorigenesis in vivo in a xenograft human colon cancer mouse model. HCT116 human colon cancer cells were subcutaneously injected into Rag1^−/−^‐immunodeficient mice. After 6 days of tumor injection, the tumor‐bearing mice were administrated with low SCT (15 mg kg^−1^ body weight), high SCT (30 mg kg^−1^ body weight), or solvent control through intraperitoneal injection at every other day for 16 days. At the endpoint of the experiments, the tumors were collected for evaluation of tumor growth and senescence induction, as well as metabolic regulations. HCT116 human colon cancer cells grew progressively in Rag1^‐/‐^ mice. However, treatment with SCT significantly inhibited tumor growth and decreased tumor weights in mice in a dose‐dependent manner (**Figure** **8**A,B). These results clearly suggest that citrate has a potent anti‐tumor capacity in vivo.

To further investigate molecular processes of citrate‐induced tumor growth in vivo, we found that citrate treatment also dramatically inhibited HCT116 tumor cell proliferation and decreased Ki‐67^+^ cell populations in tumor tissues (Figure 8C). Consistent with in vitro studies, citrate treatment significantly induced tumor cell senescence but not apoptosis in the tumor tissues (Figure 8D,E). Furthermore, citrate treatment also markedly increased the expression of cell cycle regulatory molecules P21 and P53, and phosphorylated H2AX in tumor tissues (Figure S9A, Supporting Information). In addition, citrate treatment induced excessive lipid biosynthesis and LD accumulation in tumor tissues by increasing Oil Red O^+^ cell populations and gene expression levels of lipid‐associated enzymes (Figure 8F,G). To identify signaling pathways involved in citrate‐mediated tumor suppression in vivo, we found that administration of SCT activated MAPK and mTOR signaling pathways in HCT116 tumor cells in vivo (Figure 8H). Collectively, these results clearly indicate that citrate can promote lipid metabolism and cell senescence in tumor cells, resulting in inhibition of tumor tumorigenesis and growth of colon cancer in vivo.

We further explored the translational potential of citrate for tumor treatment in a syngeneic mouse MC38 colon cancer model. Our in vitro studies have confirmed that both SCT and CA could strongly suppress the proliferation of mouse MC38 colon cancer cells (Figure S9B, Supporting Information). We then subcutaneously injected MC38 cancer cells into C57BL/6 mice. After 5 days of tumor injection, SCT was administered. We found that treatment with SCT also significantly inhibited tumor growth of MC38 in mice (Figure S9C, Supporting Information). To further dissect whether citrate treatment affects immune components and anti‐tumor immunity, we purified the immune cells from different organs and tumor tissues. We found SCT treatment markedly increased CD4^+^ T cell, B cell, and NKT cell fractions in spleens, NKT cells in lymph nodes, as well as increased Th1 and Th17, as well as granzyme B^+^, perforin^+^, and IFN‐*γ* ^+^ CD8^+^ effector T cell populations in the tumor‐infiltrating T cells (Figure S9D,E, Supporting Information). Notably, SCT treatment slightly decreased CD3^+^ cell fraction and increased IL‐4^+^ and IL‐10^+^ CD4^+^ T cell populations in the tumor‐infiltrating T cells (Figure S9E, Supporting Information). In addition, we did not find that SCT treatment significantly altered body weights, and levels of glucose, fatty acid, and citrate in serum of the MC38 tumor‐bearing mice (Data not shown). These results suggest that citrate treatment can both enhance anti‐tumor immunity and directly suppress tumor growth.

### 2.8 Citrate Synergistically Enhances Anti‐Tumor Efficacy by Chemotherapeutic Drugs In Vivo

**Figure 9:**
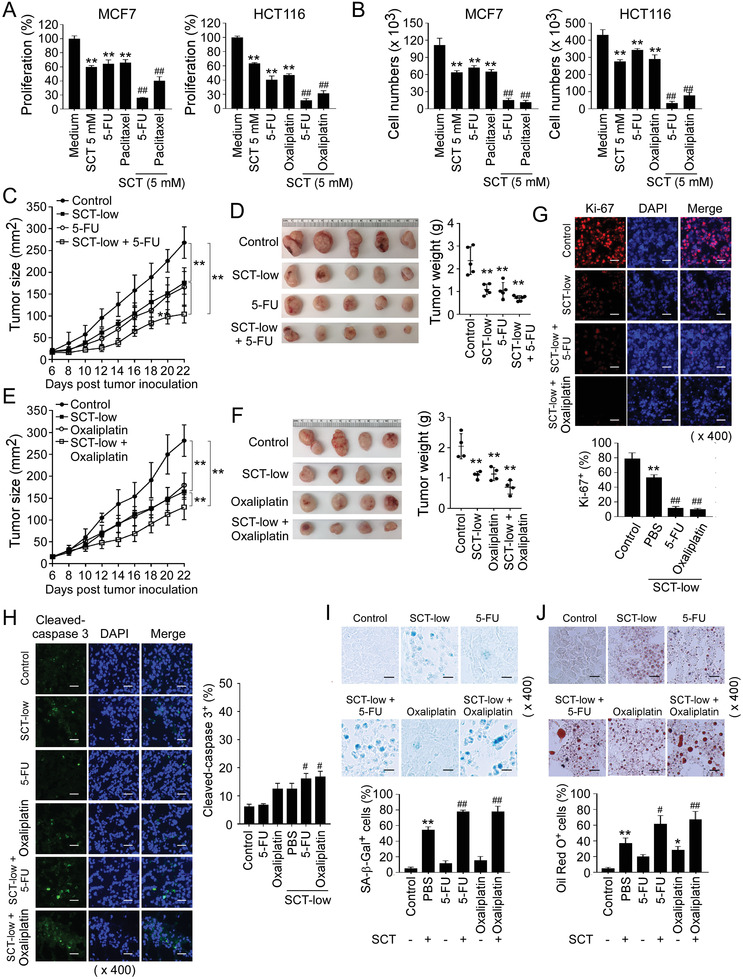
SCT administration synergistically enhances the antitumor efficacy of traditional chemotherapy. A,B) SCT treatment combined with chemotherapeutic agents showed synergistically enhanced inhibitory activity on tumor cell proliferation and growth. MCF7 and HCT116 cells were treated with SCT (5 mm) in combination with or without 5‐FU (300 µm), paclitaxel (5 µm), or oxaliplatin (50 µm). Cell proliferation and growth were determined with the MTT assay (in A) and cell numbers counting (in B), respectively. Proliferation of tumor cells with medium only was set as 100% (in A). Data shown are mean ± SD from three independent experiments. **p<0.01, compared with the medium‐only group. ^##^ *p*<0.01, compared with the citrate treatment only group. C–F) Combination with SCT treatment improved the anti‐tumor efficacy of chemotherapeutic agents in vivo in the HCT116 tumor models. HCT116 cells (4.2 × 10^6^/mouse) were subcutaneously injected into Rag1^−/−^ mice. Tumor‐bearing mice received solvent control, SCT (15 mg kg^−1^ body weight), 5‐FU (20 mg kg^−1^ body weight), or oxaliplatin (10 mg kg^−1^ body weight) by intraperitoneal injection. Tumor volumes shown in (C) and (E) were measured and presented as mean ± SD (n = 4–5 mice/group). Representative images of the xenograft tumors obtained from the indicated groups at the endpoint of the experiments (day 22) are shown in (D) and (F). In addition, histogram results shown in (D) and (F) are mean ± SD of the tumor weights from the indicated groups at the endpoint of the experiments. Each dot represents one tumor from one mouse. **p<0.01, compared with the solvent control group. G) SCT treatment combined with 5‐FU or oxaliplatin had more inhibitory activity on tumor cell proliferation than that of SCT treatment only group. Tumor frozen sections were subjected to immunofluorescence staining for Ki‐67 expression. Upper panels are representative images of Ki‐67 expression in tumor tissues from different groups. Results shown in the lower histogram are mean ± SD of Ki‐67^+^ cell fractions per high microscope field (× 400) in the tumor tissues from each group (n = 4–5 mice/group). **p<0.01, compared with the control group. ^##^ *p*<0.01, compared with the citrate treatment only group. Scale bar: 30 µm. H) SCT treatment combined with 5‐FU or oxaliplatin slightly increased apoptotic cells in tumor tissues. Cleaved caspase‐3 expression in tumor tissues was determined using the immunofluorescence assay. Left panels are representative images of cleaved caspase‐3 expression in frozen tumor tissues from different groups. Right panel is the summary of mean ± SD of cleaved caspase‐3^+^ cell fractions per high microscope field (× 400) in the tumor tissues from 4 mice of each group. ^#^ *p*<0.05, compared with the citrate‐treated only group. Scale bar: 30 µm. I,J) SCT treatment combined with 5‐FU or oxaliplatin promoted senescence induction (in I) and LD accumulation (in J) in tumor tissues than that of SCT treatment only group. SA‐*β*‐Gal and Oil Red O staining were performed in the tumor frozen tissues from different groups, respectively. Data shown in the histogram are mean ± SD of SA‐*β*‐Gal^+^/Oil Red O^+^ cell numbers per high microscope field (× 400) in the tumor tissues from 4 mice of each group. *p<0.05 and **p<0.01, compared with the control group. ^##^ *p*<0.01, compared with the citrate‐treated only group. Scale bar: 30 µm. ANOVA was performed in (A–J).

Given that citrate has potent anti‐tumor activity on tumor growth in vitro and in vivo, we questioned whether the combined use of citrate can improve anti‐tumor efficacy mediated by conventional chemotherapeutic drugs. We found that treatments with chemotherapeutic drugs 5‐fluorouracil (5‐FU) and paclitaxel in breast cancer MCF7 cells, as well as 5‐FU and oxaliplatin in colon cancer HCT116 cells, strongly suppressed tumor proliferation and growth in vitro (**Figure** **9**A,B). Furthermore, combining the drugs with SCT treatments significantly enhanced the cytotoxicity and anti‐tumor efficacy mediated by the individual drug in both MCF and HCT116 tumor cells (Figure 9A,B).

To investigate whether combination treatments of citrate with the conventional chemotherapeutic drugs can synergistically enhance chemotherapy efficacy in vivo, we first utilized the same xenograft mouse tumor model with human colon cancer in Rag1^−/−^‐immunodeficient mice as shown in Figure 8. In addition, we only used the low dose of SCT (15 mg kg^−1^) for the combination treatments. We found that treatment with 5‐FU or oxaliplatin significantly inhibited HCT116 colon cancer growth and decreased tumor weights in tumor‐bearing mice, which has similar effects as that of low dose of SCT treatment in HCT116 colon cancer (Figure 9C–F). However, combined treatments of 5‐FU or oxaliplatin with low dose SCT synergistically enhanced suppression of tumor growth and progression in HCT116 tumor‐bearing mice (Figure 9C–F). These results clearly suggest that combined use of citrate can improve anti‐tumor efficacy of conventional chemotherapeutic drugs.

To further confirm that citrate could promote lipid metabolism and induce tumor cell senescence, we determined those molecular processes in the tumor tissues. Consistent with the results shown in Figure 8, low concentration of SCT administration dramatically decreased cell proliferation (Ki‐67^+^ cell populations), and potently promoted cell senescence and LD accumulation (Figure 9G–J). SCT alone or combination with 5‐FU or oxaliplatin only induced low levels of tumor cell apoptosis in tumor tissues (Figure 9H). However, the combination of citrate with 5‐FU or oxaliplatin had more potent suppressive activity on tumor cell proliferation, induced higher levels of cell senescence and LD accumulation than that of SCT treatment only group (Figure 9G–J). These results clearly suggest that citrate sensitized tumors to chemotherapy in a synergistic fashion.

## 3 Discussion

Malignant tumors show heightened bioenergetic processes of glucose and lipid metabolism to fulfill increased energy demands for their transformation and progression.^[^ ^1^, ^2^, ^3^ ^]^ Reprograming of tumor metabolism is a novel and promising strategy for cancer treatment. Citrate is a key metabolite and critical metabolic regulator. Recent studies have shown that extracellular citrate administration displays potent inhibitory effects on tumor cells.^[^ ^12^, ^13^, ^19^, ^44^ ^]^ However, mechanisms responsible for the effects are not fully understood. In this study, we demonstrated that citrate treatment can significantly suppress tumor cell proliferation and growth in various tumor types. We also identified that citrate‐mediated tumor suppression is mechanistically due to the promotion of excessive lipid biosynthesis and cell senescence in tumor cells. Importantly, our in vivo studies clearly demonstrated that citrate administration not only dramatically inhibits tumor growth and progression, but also can synergistically enhance antitumor efficacy of the conventional chemotherapy drugs in vivo in the colon cancer models. Our studies indicate that citrate administration can rewrite tumor lipid metabolism and is an effective and promising therapeutic strategy for cancer treatment.

Citrate plays a crucial regulatory role in providing energy sources and maintaining energy homeostasis.^[^ ^16^, ^45^ ^]^ However, reduced concentrations of citrate were observed in multiple tumor tissues and patient blood and could be an indicator of cancer aggressiveness and metabolic prognosis.^[^ ^16^ ^]^ In the current study, we demonstrated overexpression of ACLY in cancer patients in various cancer types, which is negatively associated with clinical outcomes. Therefore, increasing the level of citrate in the tumor microenvironment could be a potential strategy for tumor therapy. In support of this concept, recent studies have shown that administration of high‐dose citrate or ACLY inhibition to increase the intracellular citrate induces antineoplastic effects.^[^ ^16^, ^18^, ^46^ ^]^ Furthermore, several clinical case reports demonstrated that oral administration of CA as dietary supplementation exhibits satisfying clinical improvement.^[^ ^47^, ^48^ ^]^ In this study, we further performed comprehensive studies to show that both SCT and CA of citrate can strongly inhibit tumor proliferation, growth, and functions in various types of human cancers, including breast, colon, and lung cancers. In addition, our in vivo studies using the colon cancer models demonstrated that citrate administration significantly suppresses tumor growth and progression, as well as enhance anti‐tumor immune responses in colon cancer. These studies provide more evidence that citrate is a novel target for the development of cancer therapies.

In addition to characterizing the anti‐tumor efficacy in different tumors mediated by citrate, we have also identified the mechanism responsible for citrate‐induced tumor suppression. Our current studies clearly demonstrated that both SCT and CA can initiate ATM‐associated DDR in various types of tumor cells, resulting in tumor cell senescence. Several studies from other groups have reported that SCT treatment has strong antitumor effects in various cancer cell lines by inducing apoptosis.^[^ ^49^, ^50^ ^]^ However, they used a high concentration of SCT for the studies. Furthermore, they did not consider the potentially different effects between SCT and CA administrations in their studies. In our efforts to address these issues, we compared different concentrations of SCT and CA on various types of tumor cells and found that only a high concentration of SCT treatment (above 10 mm) could promote tumor cell apoptosis. However, our studies demonstrated that low concentrations of SCT and CA significantly promote tumor cell senescence rather than apoptosis. In fact, the emerging concept of the “pro‐senescence” approach for cancer treatment has recently provoked a considerable interest because senescence impairs tumor progression and enhances the outcome of conventional anticancer therapies.^[^ ^51^, ^52^, ^53^, ^54^, ^55^ ^]^ These studies further support the rational for the development of citrate as a novel target for tumor therapy.

We also identified the molecular processes underlying citrate‐induced tumor cell senescence. Under normoxic conditions of the tumor microenvironment, the reduced citrate levels caused by the Warburg effect in tumor cells may theoretically exert feedback regulation of glycolysis.^[^ ^14^, ^18^ ^]^ However, we did not observe significant effects of SCT treatment on the expression levels of key enzymes involved in glycolysis in tumor cells. Furthermore, treatments with both SCT and CA do not significantly affect glucose uptake by tumor cells. In contrast, we found that citrate treatment induces a high level of lipid metabolism in tumor cells through the regulation of different molecular levels. First, citrate promotes gene expression of key enzymes related to cholesterol and fatty acid synthesis and catabolism in tumor cells, as well as key enzymes involved in the synthesis of CE, TAG, and phospholipids. Second, citrate treatment enhances LD accumulation in tumor cells. In addition, we identified that the promotion of excessive lipid biosynthesis in tumor cells is the cause for tumor cell senescence and growth inhibition induced by citrate. Lipid dysfunction is now well‐recognized as a common feature of cancer aggressiveness.^[^ ^3^ ^]^ However, excessive lipid accumulation may disrupt endoplasmic reticulum and induce mitochondrial dysfunctions.^[^ ^56^, ^57^ ^]^ Our studies clearly indicate that citrate can reprogram lipid metabolism in tumor cells and control tumor cell fate (Figure S10, Supporting Information). Importantly, our current study identified the molecular signaling pathways that causatively link the DNA damage, lipid metabolism, cell senescence, and suppression. Consistent with previous reports, we found that citrate treatment in tumor cells modulate ATM‐associated DNA damage, activates ERK1/2 and p38 MAPK, and mTOR signaling pathways during tumor cell senescence development.^[^ ^31^, ^32^, ^33^ ^]^ In addition, ATM‐associated DDR cooperates with MAPK and mTOR signing pathways to control hyper‐activation of lipid metabolism, which results in tumor cell senescence and growth inhibition.^[^ ^29^, ^31^, ^32^, ^33^, ^34^, ^43^, ^52^ ^]^ Collectively, our studies have identified the mechanistic basis for the development of citrate as a therapeutic target for tumor treatment.

Our studies provide another potential therapeutic strategy of combining citrate with other conventional chemotherapeutic drugs for tumor treatment. Although conventional chemotherapy has beneficial therapeutic effects in clinical applications, the major problems are therapeutic resistance and serious adverse events.^[^ ^58^ ^]^ In our current studies, we evaluated the antitumor effects of citrate treatment alone or in combination with conventional chemotherapeutic drugs, including 5‐FU or oxaliplatin in a colon cancer xenograft model. In addition to direct inhibition on tumor growth by citrate and those chemotherapeutic drugs, combination treatment of low dose SCT with 5‐FU or oxaliplatin synergistically enhances the suppressive effect on tumor growth and progression mediated by the chemotherapeutic drugs in the colon cancer models. These studies clearly suggest citrate as a promising adjuvant to conventional chemotherapy.

In summary, our study provides evidence that citrate treatment suppresses tumor cell proliferation and functions in vitro and in vivo. Mechanistically, citrate promotes excessive lipid biosynthesis, initiates DDR, and induces cell senescence in tumor cells (Figure S10, Supporting Information). In addition, our studies provide proof‐of‐concept that citrate treatment, either alone or as adjuvant combined with other cancer therapies, could be novel and effective strategies for tumor therapy.

## 4 Experimental Section

### Cell Lines

Different types of tumor cell lines (MCF7, MDA‐MB‐231, HCT116, HT29, A549, and H1299), human foreskin fibroblast (HFF), and MCF10A were all purchased from the American Type Culture Collection (ATCC). Normal lung fibroblast cell line WI‐38 was obtained from Shanghai Chinese Academy of Sciences Cell Bank (Shanghai, China). HCT116, HT29, WI‐38, and HFF were maintained in DMEM medium supplemented with 10% fetal bovine serum (FBS) and penicillin‐streptomycin. Other cell lines were cultured in RPMI‐1640 medium containing 10% FBS (all from Sigma‐Aldrich).

### Chemical Compounds

SCT tribasic dihydrate (#71 402, Sigma‐Aldrich) and CA (#C2404, Sigma‐Aldrich) were dissolved in phosphate‐buffered saline (PBS) to obtain stock solutions (1 m). Concentrated solutions of chemotherapeutic drugs paclitaxel (#T1912, Sigma‐Aldrich), 5‐FU (#F6627, Sigma‐Aldrich), and oxaliplatin (#O9512, Sigma‐Aldrich) were formulated initially in dimethyl sulfoxide (DMSO) or PBS and then diluted in RPMI medium. All stock solutions were sterilized by passing through a 0.22‐µm pore filter and stored at −20 °C for experiments.

### Cell Proliferation and Growth Assays

The MTT assay was performed according to the manufacturer's instructions. Briefly, optimized numbers of cells (3 × 10^3^ per well for MCF7, MDA‐MB‐231, H1299, A549, and MCF10A, 5 × 10^3^ per well for HCT116 and HT29, 2 × 10^3^ per well for WI‐38, and 600 per well for HFF) were seeded into 96‐well plates and exposed to varying concentrations of SCT and CA. Thereafter, 15 *μ*L of MTT solution (5 mg mL^−1^ in PBS, Sigma‐Aldrich) was added to each well. After incubation at 37 °C for 4 h, formazan crystals were dissolved in 150 *μ*L DMSO and the optical density was recorded at 570 nm. The cell proliferation was calculated as a percentage relative to the medium‐only group (set as 100%).

For growth curve assay, cells were cultured at a starting number of 5 × 10^4^/well, 2 × 10^4^/well, or 8 × 10^3^/well in 24‐well plates with the indicated concentrations of SCT or CA in the triplicate wells. Cell growth was evaluated at different time points by counting cell numbers.

EdU (5‐Ethynyl‐2′‐deoxyuridine) assay was performed using the BeyoClick EdU Cell Proliferation Kit (#C0075, Beyotime) following the manufacturer's instructions. In brief, different numbers of tumor cells (3 × 10^4^ per well for MCF7, H1299, and A549, and 4 × 10^4^ per well for HCT116) were seeded into 48‐well plates and exposed to the indicated concentrations of SCT for 24 h. After incubation with 10 µm EdU at 37  °C for 2 h, cells were fixed with 4% paraformaldehyde for 15 min and then permeabilized with 0.3% Triton X‐100 for 15 min. Treated cells were then stained with Click Addictive Solution and Hoechst 33 342, and observed with a fluorescence microscope.

### Cell Cycle Analysis

Tumor cells were seeded in the 6‐well plates (2 × 10^5^ per well) and treated with SCT treatment for 48 h. Tumor cells were fixed with 70% ethanol overnight at 4 °C, and then incubated with propidium iodide (10 µg mL^−1^) and RNase A (100 µg mL^−1^) for 30 min. All of the stained cells were analyzed by flow cytometry (CytoFLEX, Beckman) and data analysis with the ModFit LT 5.0 software (Verity Software House).

### SA‐*β*‐Gal Staining

The SA‐*β*‐Gal staining was performed as described previously.^[^ ^29^, ^39^, ^42^ ^]^ Co‐cultured tumor cells or the frozen tissue sections from in vivo experiments (4–8 µm) were washed in PBS, fixed in 4% formaldehyde, and incubated overnight at 37 °C with freshly prepared staining solution. The frozen sections were restored up to room temperature for 30 min before staining. Five sections were analyzed for each mouse. The blue‐stained senescent cells were counted manually under a microscope and calculated as a percentage of total cell numbers.

For some experiments, SA‐*β*‐Gal^+^ tumor cells were determined with the pretreatment of cells the following inhibitors: ATM inhibitor KU55933 (5 *μ* m, #3544, Tocris Bioscience), MAPK inhibitors U0126 (4 *μ* m, #19–147, Sigma‐Aldrich) and SB203580 (5 *μ* m, #AG‐CR1‐0030, AdipoGen), mTOR inhibitor rapamycin (50 nm, #R0395, Sigma‐Aldrich), FASN inhibitor C75 (5 *μ* m, #10 005 270, Cayman Chemical), HMGCR inhibitor simvastatin (1 *μ* m, #10 010 344, Cayman Chemical), IDI1 inhibitor 25‐HC (0.25 µg mL^−1^, #11 097, Cayman Chemical), and ACAT inhibitor avasimible (1 *μ* m, #18 129, Cayman Chemical).

### Glucose Uptake Assay

Glucose uptake was determined following 15‐min incubation of tumor cells with a fluorescent D‐glucose analog 2‐[(7‐nitrobenz‐2‐oxa‐1,3‐diazol‐4‐yl) amino]‐2‐deoxy‐D‐glucose (2‐NBDG) (Cayman Chemical), as previously described.^[^ ^32^ ^]^ Tumor cells were pretreated with different concentrations of SCT or CA for 24 h, and then starved by cultured in glucose‐free RPMI 1640 medium (Gibco) with 2% FBS for an additional 30 min. After 15‐min incubation with 2‐NBDG (100 µm), the cells were harvested and analyzed with a FACS Calibur flow cytometer (BD Bioscience).

### Oil Red O Staining

Oil Red O staining was performed as described previously.^[^ ^38^ ^]^ The treated tumor cells were washed in PBS (pH 7.2), fixed with 4% formaldehyde for 30 min, and treated with 60% isopropanol for 2–5 min. Cells were further stained with freshly prepared Oil‐Red O staining solution (Sigma‐Aldrich) in isopropanol (60%) for 5 min, followed by a quick rinse with isopropanol (60%). The stained cells were then washed with H_2_O thoroughly and LDs in tumor cells were evaluated using a light microscope.

### Quantification of Citric Acid, Free Fatty Acid, and Total Cholesterol

CA, free fatty acid, and total cholesterol productions were measured using the detection kits (#BC2155 Solarbio, MAK044 Sigma, and #BC1985 Solarbio, respectively) following the protocols provided by the manufacturers. Briefly, intracellular CA, free fatty acid, or cholesterol from different types of tumor cells were harvested with respective extracting solutions from cell pellets after cell ultrasonication or grinding. The absorbances were measured by a microplate reader at 545 or 500 nm. All experiments were performed three times and the data were normalized by the cell numbers.

### Western Blot Analysis

Tumor cells were cultured in the medium with indicated concentrations of citrate for different time points. Whole cell lysates were prepared for western blot analysis. The antibodies used in western blot analyses were as following: anti‐caspase‐3 (#9662), anti‐cleaved caspase‐3 (Asp175, #9664), anti‐Mcl‐1 (#5453), anti‐ERK (#4695), anti‐phospho‐ERK (Thr180/Tyr182, #9101), anti‐p38 (#9212), anti‐phospho‐p38 (Thr180/Tyr182, #4511), anti‐mTOR (#2983), anti‐phospho‐mTOR (Ser2448, #2971), anti‐P70S6K (#2708), anti‐phospho‐P70S6K (Thr421/Ser424, #9204), anti‐4EBP1 (#9644), anti‐phospho‐4EBP1 (Thr37‐46, #2855), and anti‐GAPDH (#2118) rabbit polyclonal antibodies (from Cell Signaling Technology), as well as anti‐Bax (#A12009) and anti‐Bcl‐2 (#A0208) rabbit polyclonal antibodies (from ABclonal Technology). The dilution for primary antibody was 1:1000.

### Flow Cytometry Analysis

For cell apoptosis assay, tumor cells were collected after citrate treatment and detected with PE‐Annexin V Apoptosis Detection Kit (BD Biosciences). The markers on tumor cells or immune cells were examined by the flow cytometry analysis after surface staining or intracellular staining indicated antibodies. The antibodies for DNA damage markers included: anti‐phospho‐ATM (Ser1981, #13 050), anti‐phospho‐H2AX (Ser139/Tyr142, #5438), anti‐phospho‐CHK2 (Thr68, #2661), and anti‐phospho‐53BP (Ser25/29, #2675) (1:200 dilution, Cell Signaling Technology). The antibodies for immune cells included: anti‐CD3 (17A2), anti‐CD4 (GK1.5), anti‐CD8 (53–6.7), anti‐CD19 (6D5), anti‐NK1.1 (PK136), anti‐IFN‐*γ* (XMG1.2), anti‐IL‐4 (11B11, BD), anti‐IL‐10 (JES5‐16E3), anti‐IL‐17A (TC11‐18H10.1), anti‐granzyme B (GB11), and anti‐perforin (S16009A), which were all purchased from Biolegend. All stained cells were analyzed on a Guava EasyCyte Plus Flow Cytometer (Millipore) and data were analyzed with the FlowJo software (Tree Star).

### Indirect Immunofluorescence Staining

Citrate‐treated tumor cells were incubated with primary rabbit anti‐human antibodies, including anti‐p53 (#2524), anti‐p21 (#2947), and anti‐p16 (#92 803) (Cell Signaling Technology), or antibodies as described above. The dilution of these antibodies was 1:100 for the studies. Tumor cells were then incubated with Alexa Fluor568‐conjugated anti‐rabbit or Alexa Fluor594‐conjugated anti‐mouse secondary antibodies (#A11011, #A21203, Invitrogen), and were further counterstained with 4’,6 diamidino‐2‐phenylindole (DAPI, Invitrogen).

### Quantitative Real‐Time PCR Analysis

Total RNA was extracted from tumor cells or tissues using Trizol reagent (Invitrogen) and transcribed to cDNA using a cDNA Reverse Transcription Kit (Invitrogen) following the manufacturer's instructions. Gene expressions were determined by real‐time qPCR and the mRNA levels in each sample were normalized to the relative quantity of the housekeeping *β*‐actin gene expression. All experiments were performed in triplicate. The specific primers used for tumor cells are listed in Table S1, Supporting Information.

### In Vivo Therapeutic Tumor Xenograft Studies

C57BL/6 and Rag1^−/−^‐immunodeficient mice (6–8 weeks old) were purchased from The Jackson Laboratory and maintained in the institutional animal facility. All animal studies have been approved by the Institutional Animal Care Committee at Saint Louis University (Protocol No. 2411). Human colon cancer HCT116 cells (4.2 × 10^6^/mouse) were subcutaneously injected into Rag1^−/−^ immunodeficient mice. After 6 days of tumor injection, SCT alone (15 or 30 mg kg^−1^) or in combination with chemotherapeutic drugs (20 mg kg^−1^ 5‐FU or 10 mg kg^−1^ oxaliplatin) were intraperitoneally injected every other day for 16 days. Five to ten mice were included in each group. Tumor volumes were calculated by caliper measurements using the formula length × width. Tumor‐bearing mice were sacrificed at the endpoint of the experiment and tumors were isolated and weighted. Frozen sections were prepared for immunohistochemical staining, SA‐*β*‐Gal, and Oil Red O staining as described above. The primary antibodies for immunohistochemical staining include anti‐Ki67 (#9129, 1:1000), anti‐cleaved caspase‐3 (#9664, 1:400), anti‐p53 (#2524, 1:100), anti‐p21 (#2947, 1:100), and anti‐phospho‐H2AX (#5438, 1:100) with the indicated dilution, respectively (Cell Signaling Technology). The tumor tissues were also lysed for western blot and real‐time qPCR assays.

For the syngeneic mouse colon cancer model, mouse colon cancer MC38 cells (0.5 × 10^6^/mouse) were subcutaneously injected into C57BL/6 mice. After 5 days of tumor injection, SCT (60mg kg^−1^ body weight) was intraperitoneally injected at every other day for twice. From day 9, SCT (60mg kg^−1^ body weight) was intratumor injected every day to the end of the experiment. Four mice were included in each group. Tumor size was measured and calculated as above. Blood, spleens, and tumors were harvested at the end of each experiment. The immune cells from different organs and tumor tissues were isolated for subsequent phenotypic and functional analyses.

### Data Acquisition and Bioinformatics Analysis

The RNA‐seq HTSeq‐FPKM data (level 3) of eight cancer types were downloaded from TCGA (https://portal.gdc.cancer.gov/). The mRNA expressions of ACLY were extracted and compared between tumor and normal tissues by the R/Bioconductor package of limma. Survival analysis was performed using a web‐tool Kaplan–Meier Plotter (http://www.kmplot.com/) with up to 180 months of follow‐up information and the samples were grouped by the auto‐selected best cutoff.^[^ ^59^ ^]^

### Statistical Analysis

Statistical analysis was performed with GraphPad Prism5 software. Data were expressed as mean ± standard deviation (SD). For multiple group comparison for in vivo studies, a one‐way analysis of variance (ANOVA) was used, followed by the Dunnett's test for comparing experimental groups against a single control. For a single comparison between two groups, paired Student's *t*‐tests were used. Nonparametric *t*‐test was chosen if the sample size was too small and did not fit the Gaussian distribution. For patient survival analysis, the log‐rank test was used to determine the statistical significance.

## Conflict of Interest

The authors declare no conflict of interest.

## Author Contributions

Y.Z., Q.S., and G.P.: designed research, analyzed data, prepared figures, and wrote the paper. Y.Z., X.L., F.S., L.H., A.G., and W.L.: performed experiments. D.H.: advised the design of research and discussed the manuscript.

## Supporting information

Supporting Information

## Data Availability

The RNA‐seq HTSeq‐FPKM data (level 3) of eight cancer types were downloaded from The Cancer Genome Atlas (TCGA, https://portal.gdc.cancer.gov/). The mRNA expressions of ACLY were extracted and compared between tumor and normal tissues by the R/Bioconductor package of limma. Survival analysis was performed using a web‐tool Kaplan–Meier Plotter (http://www.kmplot.com/) with up to 180 months of follow‐up information and the samples were grouped by the auto‐selected best cutoff (52).
