# Supplementary material for: Citrate Promotes Excessive Lipid Biosynthesis and Senescence in Tumor Cells for Tumor Therapy
Source: Adv Sci (Weinh). 2021 Nov 7;9(1):2101553. doi: 10.1002/advs.202101553 (PMC8728847; doi:10.1002/advs.202101553)
Supplement: Supplementary file 1 — Supporting Information [file ADVS-9-2101553-s001.pdf]

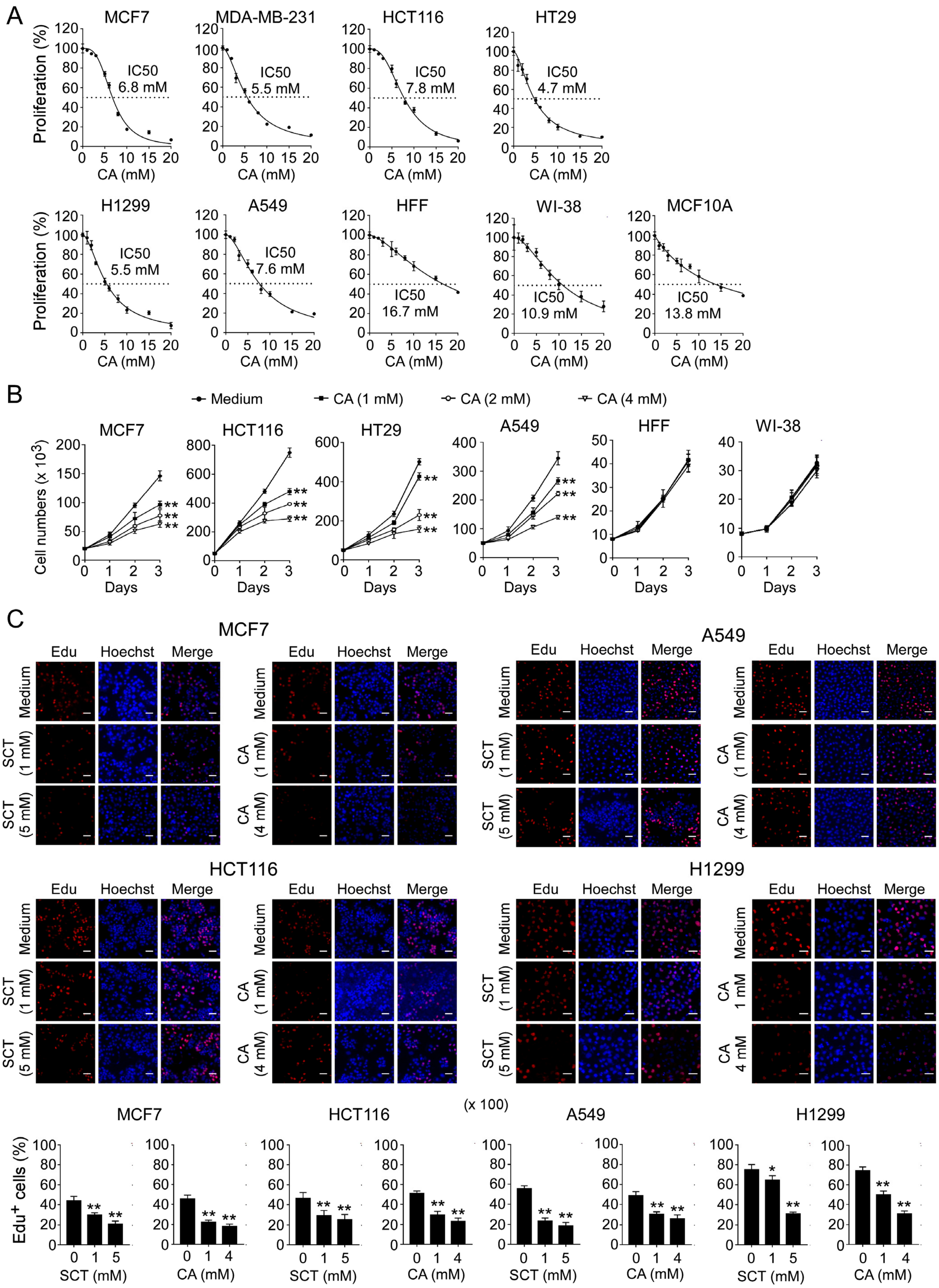

A
MCF7
MDA-MB-231
HCT116
HT29
H1299
A549
HFF
WI-38
MCF10A
Proliferation (%)
CA (mM)
IC50
6.8 mM
5.5 mM
7.8 mM
4.7 mM
5.5 mM
7.6 mM
16.7 mM
10.9 mM
13.8 mM
B
Medium
CA (1 mM)
CA (2 mM)
CA (4 mM)
Cell numbers (x 10^3)
Days
C
Edu
Hoechst
Merge
SCT (1 mM)
SCT (5 mM)
CA (1 mM)
CA (4 mM)
CA 1 mM
CA 4 mM
(x 100)
Edu$^+$ cells (%)
SCT (mM)

Figure S1. Citric acid suppresses tumor cell growth and proliferation.

(A) Multiple cancer cell lines were treated with the indicated different doses of CA for 24 hours and cell proliferation measured by the MTT assay. The normal HFF, WI-38 and MCF10A cells served as controls. The values of cell proliferation are shown as mean ± SD from six repeated wells. IC50 value for each cell type is a representative of three independent experiments. **(B)** Different types of tumor cells and normal HFF and WI-38 cells were treated with/without of the indicated concentrations of CA. The cell growth was evaluated at different time points using the cell number counting assay. Data shown are mean ± SD from three independent experiments. $^{**}p<0.01$, compared with the medium only group. **(C)** SCT or CA treatment significantly suppressed proliferation in different types of tumor cells. Tumor cells were treated with the indicated concentrations of SCT or CA for 24 hours and cell proliferation was determined using the Edu cell proliferation assay. Scale bar: 100 μm. Data shown in histograms are mean ± SD from three experiments with similar results. $^{*}p<0.05$ and $^{**}p<0.01$, compared with the medium only group. One-way analysis of variance (ANOVA) was performed in (B) and (C).

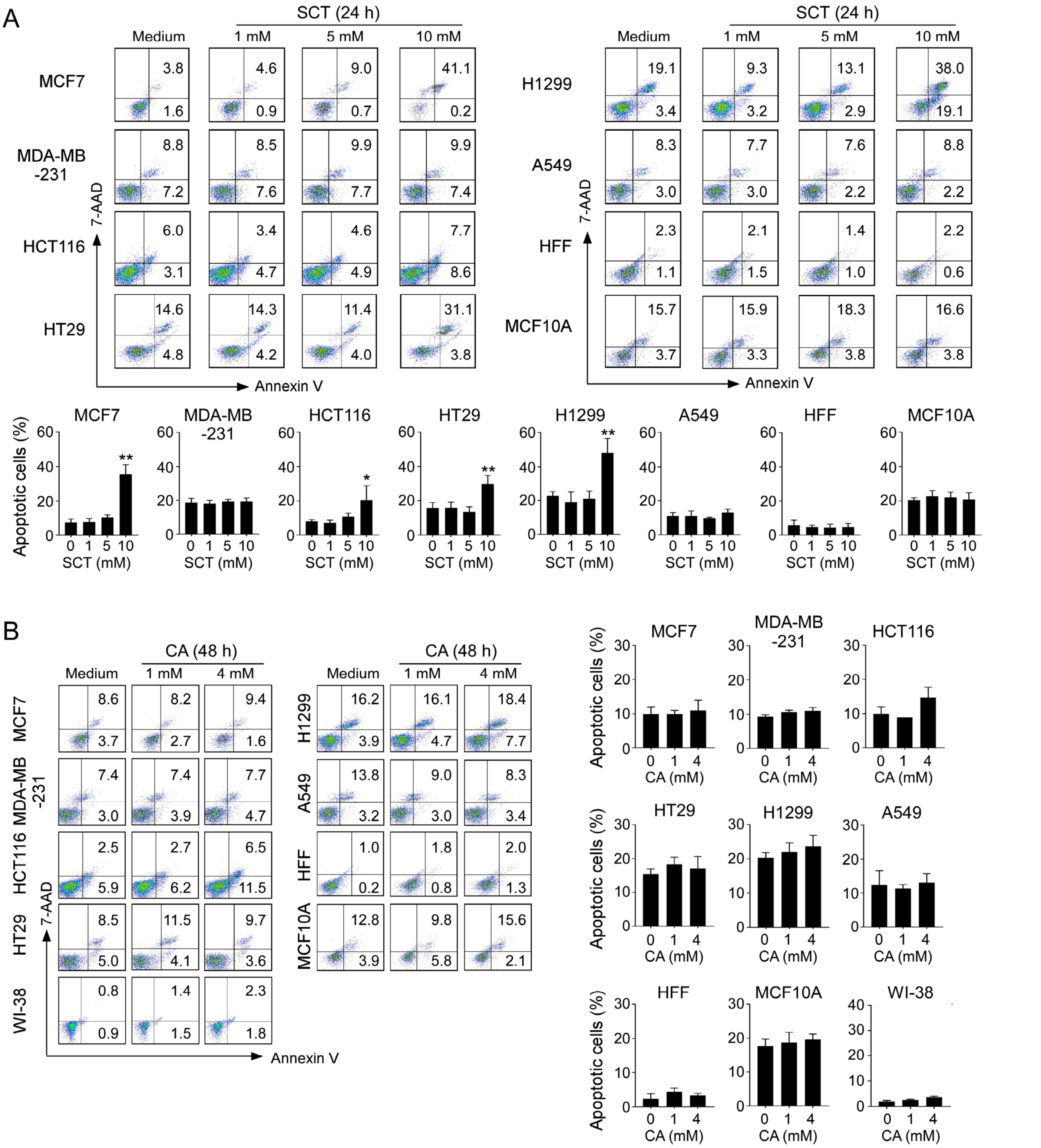

Figure S2. Low doses of citrate do not induce tumor cell apoptosis.
(A) and **(B)** Different types of tumor cells were treated with the indicated concentrations of SCT for 24 hours (in A) or with CA for 48 hours (in B). Normal HFF, WI-38 and MCF10A cells included as controls. Apoptotic cells were analyzed with the flow cytometry after Annexin V and 7-AAD double staining. Data shown in histograms are mean ± SD from three experiments with similar results. *p<0.05 and **p<0.01, compared with the medium only group using ANOVA analysis.

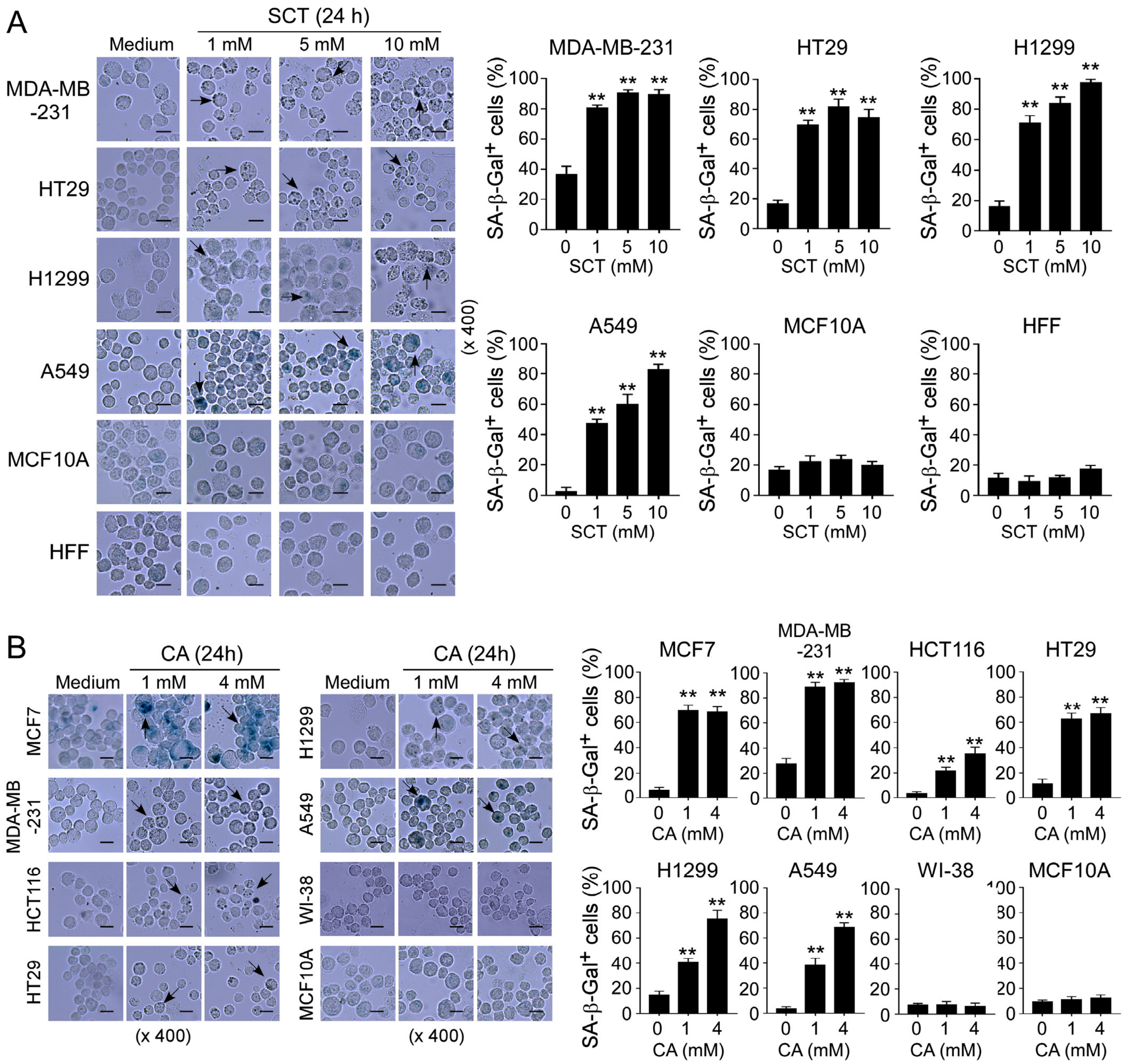

Figure S3. DNA damage response is involved in citrate-induced tumor cell senescence.

(A) and **(B)** Treatments with SCT or CA induced cell senescence in tumor cells but not in normal cells. Different types of tumor cells or normal control HFF, WI-38 and MCF10A cells were treated with the indicated concentrations of SCT (in A) or CA (in B) for 24 hours, and then stained for SA-β-Gal. SA-β-Gal$^+$ cells were identified as dark blue granules as indicated by the arrows. Data shown in histograms are mean ± SD from three independent experiments. **p<0.01, compared with the medium only group with ANOVA analysis. Scale bar: 30 μm.

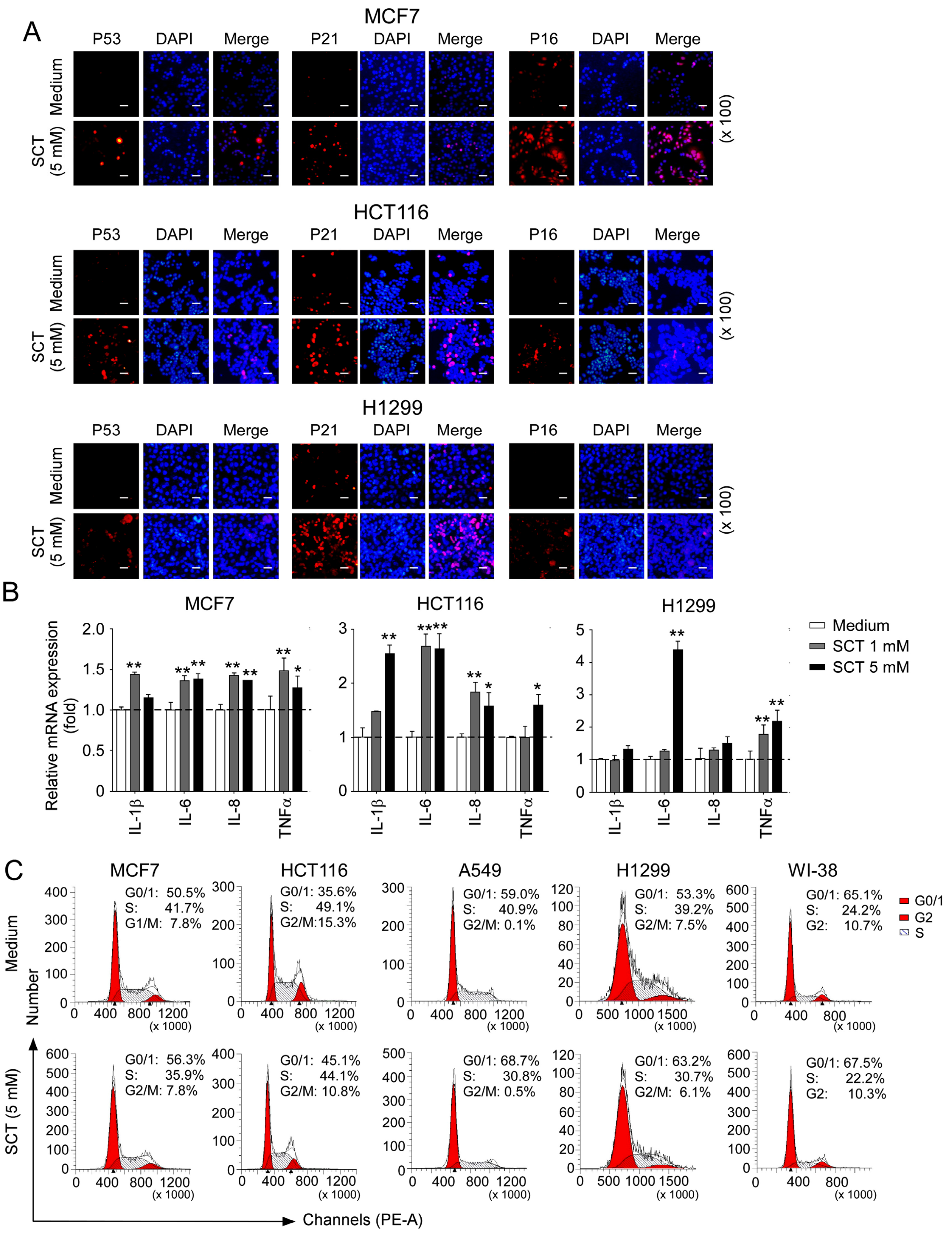

A
MCF7
P53
DAPI
Merge
P21
P16
Medium
SCT (5 mM)
(x 100)
HCT116
H1299
B
Relative mRNA expression (fold)
IL-1β
IL-6
IL-8
TNFα
Medium
SCT 1 mM
SCT 5 mM
C
MCF7
HCT116
A549
H1299
WI-38
G0/1: 50.5%
S: 41.7%
G1/M: 7.8%
G0/1: 35.6%
S: 49.1%
G2/M:15.3%
G0/1: 59.0%
S: 40.9%
G2/M: 0.1%
G0/1: 53.3%
S: 39.2%
G2/M: 7.5%
G0/1: 65.1%
S: 24.2%
G2: 10.7%
G0/1: 56.3%
S: 35.9%
G2/M: 7.8%
G0/1: 45.1%
S: 44.1%
G2/M: 10.8%
G0/1: 68.7%
S: 30.8%
G2/M: 0.5%
G0/1: 63.2%
S: 30.7%
G2/M: 6.1%
G0/1: 67.5%
S: 22.2%
G2: 10.3%
G0/1
G2
S
Number
(x 1000)
Channels (PE-A)

Figure S4. SCT treatment promotes cell cycle arrest and inflammatory cytokine production in senescent tumor cells.

(A) Immunofluorescence staining showed upregulation of cell cycle regulatory molecules P53, P21 and P16 in tumor cells after culture with 5 mM SCT for 48 hours. Scale bar: 100 μm. DAPI was used to stain cell nuclei. **(B)** Tumor cells were treated with indicated concentrations of SCT for 24 hours and mRNA expression of each cytokine was determined by the real-time PCR. Expression levels of each gene were normalized to β-actin expression level and adjusted to the levels in tumor cells treated with medium only (served as 1). Data shown are mean ± SD from three independent experiments with similar results. *p<0.05 and **p<0.01, compared with the medium only group with ANOVA analysis. **(C)** SCT treatment induced cell cycle arrest in G0/G1 phases in tumor cells but not in normal WI-38 cells. Cells were treated with 5 mM SCT for 48 hours and cell cycle distributions were measured by the flow cytometry using PI staining. Results shown are representatives of three independent experiments.

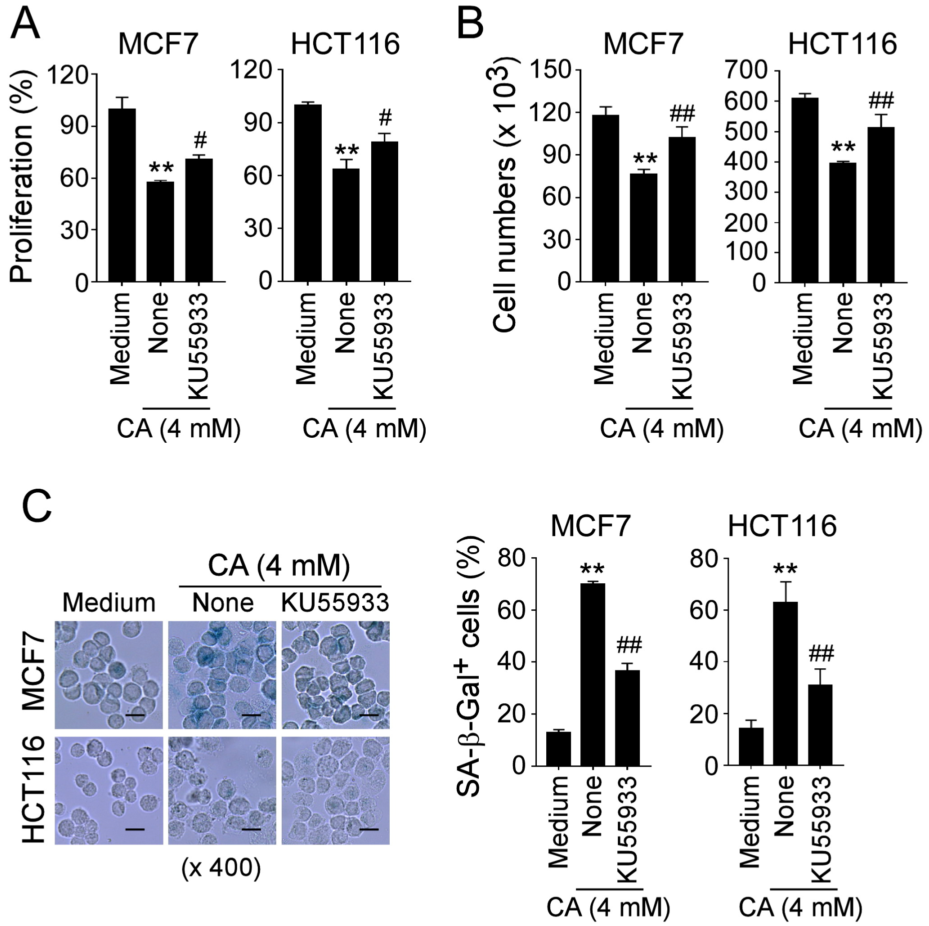

Figure S5. DNA damage response is involved in citrate-induced tumor cell senescence.
(A) and **(B)** Inhibition of ATM signaling reversed CA-induced suppression of tumor cell proliferation and growth. MCF7 and HCT116 cells were pretreated with ATM inhibitor KU55933 (5 μM) for 24 hours and then cultured with CA (4 mM) for 48 hours. Cell proliferation and growth were determined with the MTT assay (in A) and cell numbers counting (in B), respectively. Proliferation of tumor cells with medium only served as 100% (in A). Data shown are mean ± SD from three independent experiments. $^{**}p<0.01$, compared with the medium only group. $^{\#\#}p<0.01$, compared with the CA treatment only group. **(C)** Blockage of ATM signaling prevented CA-induced tumor cell senescence. Cell treatment and procedure were identical to (A) and (B). Senescent cell populations were determined using the SA-β-Gal staining. Data shown in histograms are mean ± SD from three independent experiments. $^{**}p<0.01$, compared with the medium only group. $^{\#\#}p<0.01$, compared with the citrate treatment only group. Scale bar: 30 μm. ANOVA analysis was performed in (A)-(C).

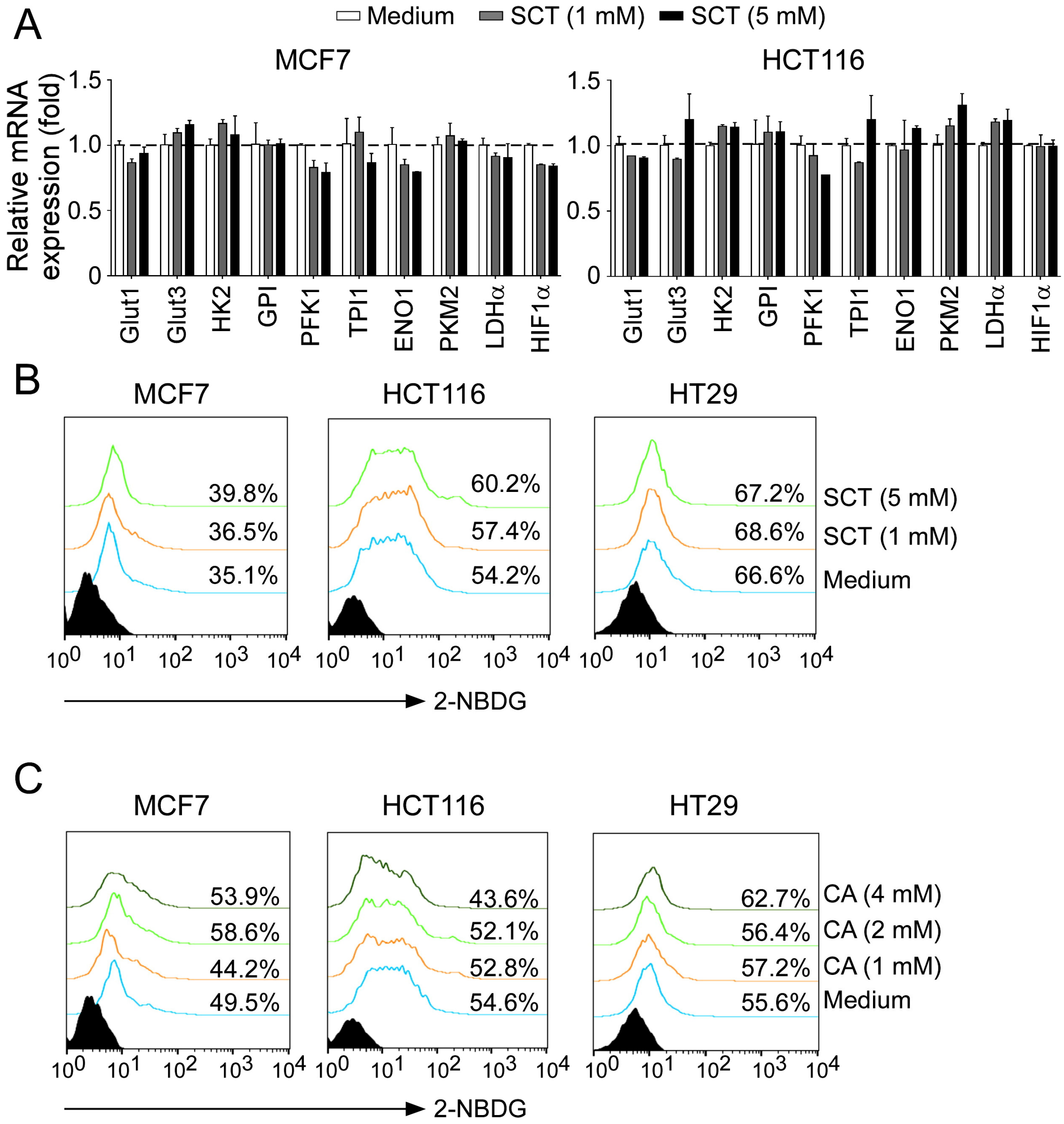

Figure S6. Citrate treatment does not affect glycolysis in tumor cells.

(A) Gene expression levels of glucose transporters (Glut1 and Glut3) and the key enzymes involved in glycolysis (HK2, GPI, PFK1, TPI, ENO1, PKM2, and LDHα) and HIF1a in MCF7 and HCT116 tumor cells were determined after coculture with SCT for 24 hours. Total RNA was isolated from the treated tumor cells and gene expression analyzed by real-time qPCR. Expression levels of each gene were normalized to β-actin expression level and adjusted to the levels in tumor cells treated with medium only (served as 1). Data shown are mean ± SD from three independent experiments with similar results. **(B)** and **(C)** Citrate treatment did not suppress glucose uptake capacity in tumor cells. Three types of tumor cells were treated with SCT (in B) or CA (in C) for 24 hours. The glucose uptake capacity in tumor cells was determined by the flow cytometry after 15 min incubation of 2-NBDG. Results shown are representatives of three independent experiments.

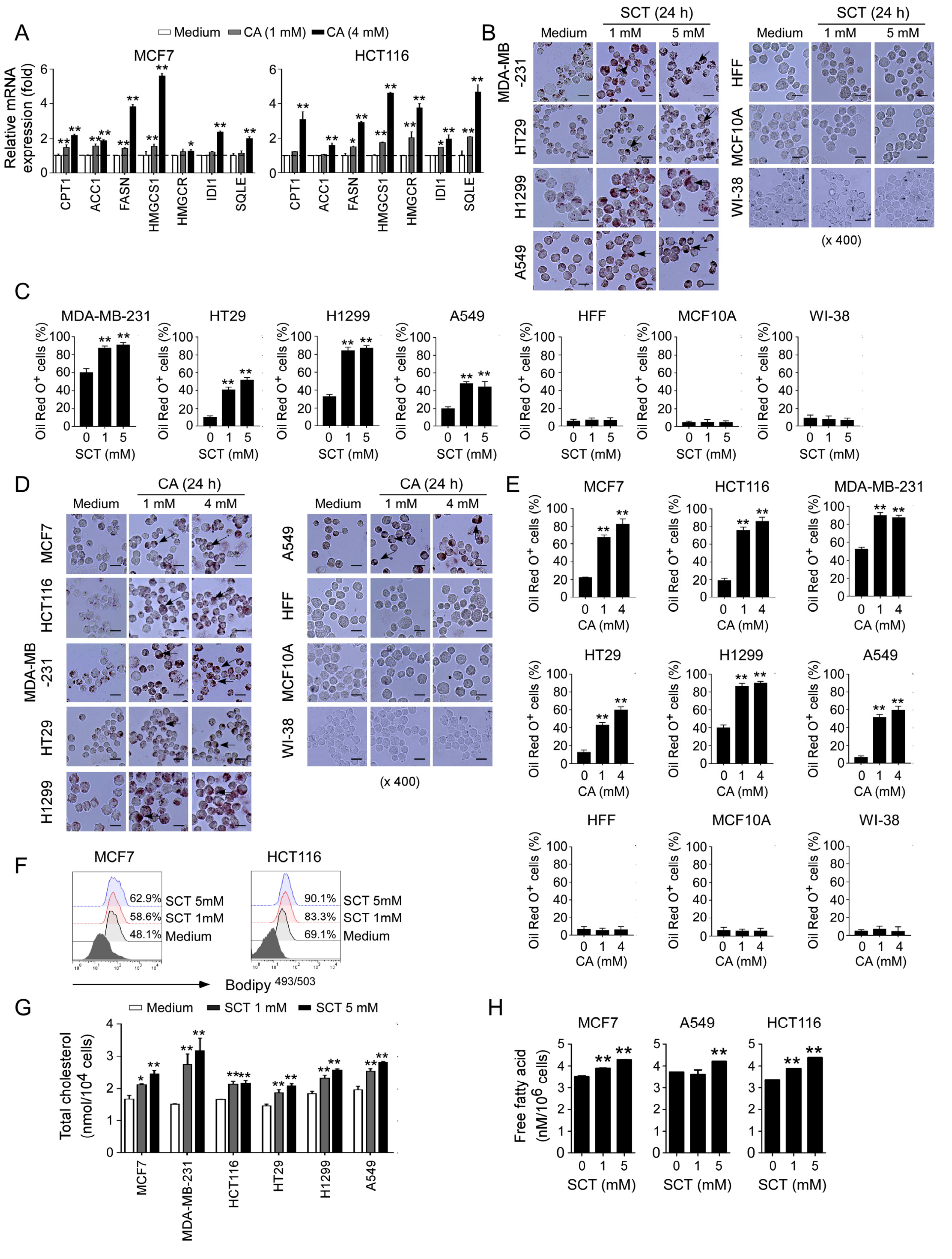

A
Medium
CA (1 mM)
CA (4 mM)
MCF7
HCT116
Relative mRNA expression (fold)
CPT1
ACC1
FASN
HMGCS1
HMGCR
IDI1
SQLE
B
SCT (24 h)
Medium
1 mM
5 mM
MDA-MB-231
HT29
H1299
A549
HFF
MCF10A
WI-38
(x 400)
C
MDA-MB-231
HT29
H1299
A549
HFF
MCF10A
WI-38
Oil Red O+ cells (%)
SCT (mM)
D
CA (24 h)
Medium
1 mM
4 mM
MCF7
HCT116
MDA-MB-231
HT29
H1299
A549
HFF
MCF10A
WI-38
(x 400)
E
MCF7
HCT116
MDA-MB-231
HT29
H1299
A549
HFF
MCF10A
WI-38
Oil Red O+ cells (%)
CA (mM)
F
MCF7
62.9% SCT 5mM
58.6% SCT 1mM
48.1% Medium
HCT116
90.1% SCT 5mM
83.3% SCT 1mM
69.1% Medium
Bodipy 493/503
G
Medium
SCT 1 mM
SCT 5 mM
Total cholesterol (nmol/10^4 cells)
MCF7
MDA-MB-231
HCT116
HT29
H1299
A549
H
MCF7
A549
HCT116
Free fatty acid (nM/10^6 cells)
SCT (mM)

Figure S7. Citric acid promotes lipid biosynthesis and cell senescence in tumor cells.
(A) Increased gene expression of key enzymes in cholesterol synthesis (HMGCR, HMGCS1, SQLE, and IDI1), as well as fatty acid oxidation (CPT-1) and synthesis (ACC1 and FASN) in MCF7 and HCT116 tumor cells was induced by the indicated concentrations of CA for 24 hours. Total RNA was isolated from the treated tumor cells and gene expression analyzed by real-time qPCR. Expression levels of each gene were normalized to β-actin expression level and adjusted to the levels in tumor cells treated with medium only (served as 1). Data shown are mean ± SD from three independent experiments with similar results. *p<0.05 and **p<0.01, compared with the medium only group. **(B)** and **(C)** Accumulated LDs in tumor cells but not control normal cells were induced by SCT treatment. Different types of tumor cells and normal control HFF, WI-38 and MCF-10A cells were cultured in the presence of indicated concentrations of SCT for 24 hours and then stained for Oil Red O. The Oil Red O^+ cells were identified with red granules as indicated by the arrows. Data shown in (C) are means ± SD from three independent experiments. **p<0.01, compared with the medium only group. Scale bar: 30 μm. **(D)** and **(E)** CA treatment promoted LD accumulation in tumor cells. Tumor cells and normal control cells were cultured in the presence of indicated concentrations of CA for 24 hours and then stained for Oil Red O. The Oil Red O^+ cells were identified with red granules as indicated by the arrows. Data shown in (E) are means ± SD from three independent experiments. **p<0.01, compared with the medium only group. Scale bar: 30 μm. **(F)** MCF7 and HCT116 cells were treated with SCT for 48 hours and LD levels in the cultured cells were determined by the flow cytometry after incubation with Bodipy493/503 for 15 minutes. Results shown are representatives of three independent experiments. **(G)** and **(H)** Citrate treatment induced production of intracellular total cholesterol (in G) and free fatty acid (in H) in tumor cells. Different types of tumor cells were treated with SCT for 48 hours, and total cholesterol (in G) and free fatty acid (in H) in tumor cells were determined using respective assay kits. *p<0.05 and **p<0.01, compared with the medium only group. ANOVA was performed in (A), (C), (E), (G) and (H).

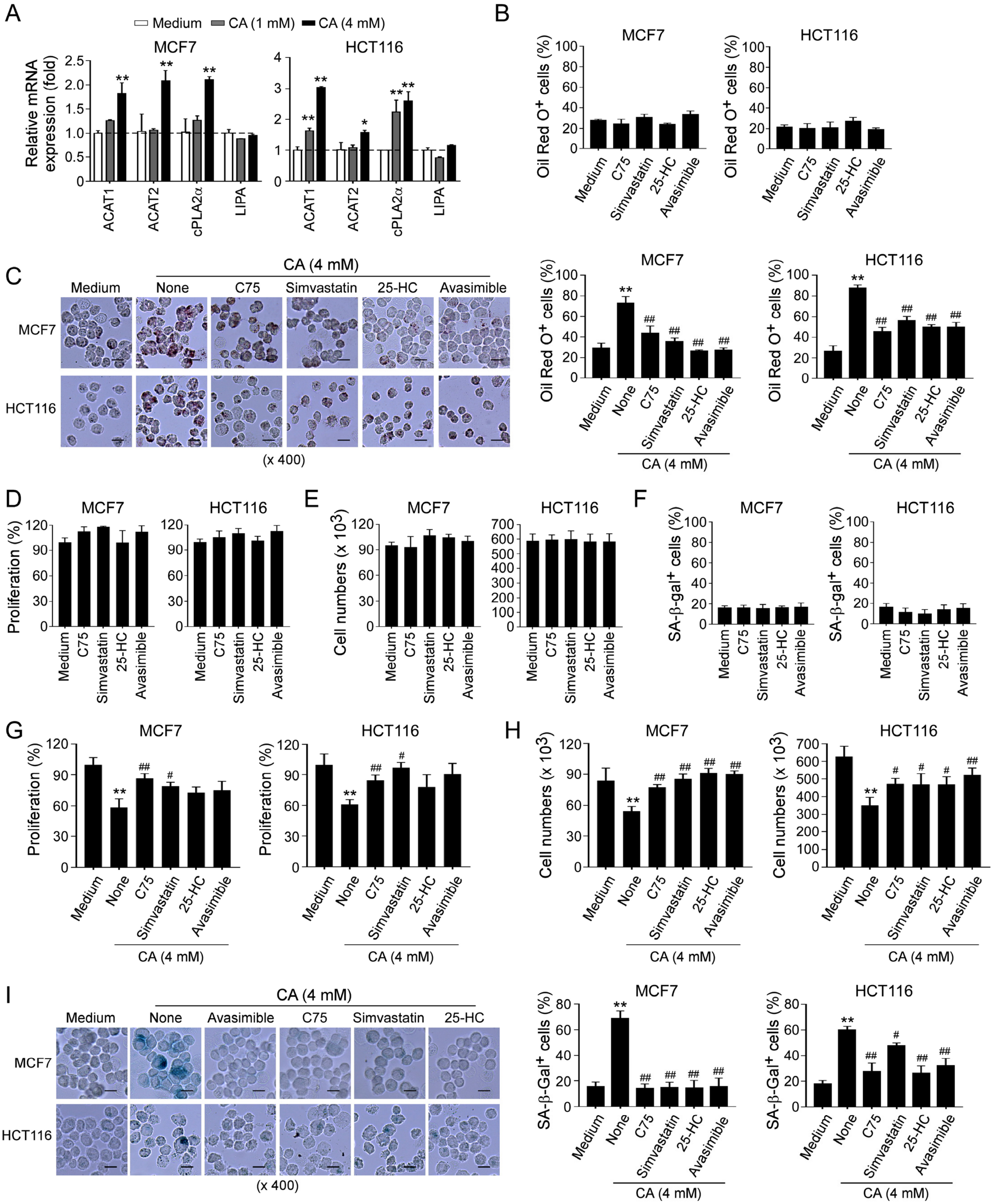
A
Medium
CA (1 mM)
CA (4 mM)
MCF7
HCT116
Relative mRNA expression (fold)
ACAT1
ACAT2
cPLA2α
LIPA
B
Oil Red O+ cells (%)
Medium
C75
Simvastatin
25-HC
Avasimible
C
CA (4 mM)
None
(x 400)
D
Proliferation (%)
E
Cell numbers (x 10³)
F
SA-β-gal+ cells (%)
G
H
I
SA-β-Gal+ cells (%)

Figure S8. Citric acid-induced lipid biosynthesis is responsible for cell senescence in tumor cells.
(A) CA treatment upregulated gene expression levels of key enzymes (ACAT1, ACAT2 and cPLA2α) involved in LD formation, but not hydrolase LIPA in tumor cells. MCF7 and HCT116 cells were treated with CA for 24 hours and mRNA expression levels of each gene were determined by the real-time qPCR. The expression level was normalized to β-actin expression and adjusted to the levels in tumor cells with medium only. Data are means ± SD from three independent experiments with similar results. *p<0.05 and **p<0.01, compared with the tumor cells in respective medium only group. **(B)** Treatments with lipid metabolism inhibitors only did not promote LD accumulation in tumor cells. MCF7 and HCT116 cells were treated with the pharmaceutical inhibitors for lipid synthesis for 24 hours, including C75 (5 μM), simvastatin (1 μM), 25-HC (0.25 μg/ml), or avasimible (1 μM), respectively. Oil Red O staining on tumor cells were performed. Data shown are mean ± SD from three independent experiments with similar results. **(C)** Blockage of the lipid synthesis reversed CA-induced LD accumulation in tumor cells. MCF7 and HCT116 cells were pretreated with the pharmaceutical inhibitors for lipid synthesis for 24 hours, including C75 (5 μM), simvastatin (1 μM), 25-HC (0.25 μg/ml), or avasimible (1 μM), respectively. Tumor cells were then cultured in the presence of CA (4 mM) for an additional 24 hours and stained for Oil Red O. Data shown in the right panels are mean ± SD from three independent experiments with similar results. **p<0.01, compared with the medium only group. ##p<0.01, compared with the citrate treatment only group. Scale bar: 30 μm. **(D)** to **(F)** Treatment with lipid metabolism inhibitors (C75, simvastatin, 25-HC, and avasimible) only neither suppressed tumor cell proliferation and growth, nor promoted tumor cell senescence. Cell treatment and procedures were identical to (B). Cell proliferation and growth were determined with the MTT assay (in D) and cell numbers counting (in E), respectively. Proliferation of tumor cells with medium only served as 100% (in D). Senescent cell populations were determined using the SA-β-Gal staining (in F). Data shown are mean ± SD from three independent experiments. **(G)** and **(H)** Inhibition of the lipid synthesis blocked CA-induced suppressive activity on tumor cell proliferation and growth. Cell treatment and procedure were identical to (C). Cell proliferation and growth were determined with the MTT assay (in G) and cell numbers counting (in H), respectively. Proliferation of tumor cells with medium only served as 100% (in G). Data shown are mean ± SD from three independent experiments. **p<0.01, compared with the medium only group. #p<0.05 and ##p<0.01, compared with the citrate treatment only group. **(I)** Blockage of the lipid synthesis prevented CA-induced tumor cell senescence. Cell treatment and procedure were identical to (C). Senescent cell populations were determined using the SA-β-Gal staining. Data shown in histograms are mean ± SD from three independent experiments. **p<0.01, compared with the medium only group. ##p<0.01, compared with the citrate treatment only group. Scale bar: 30 μm. ANOVA was performed in (A)-(I).

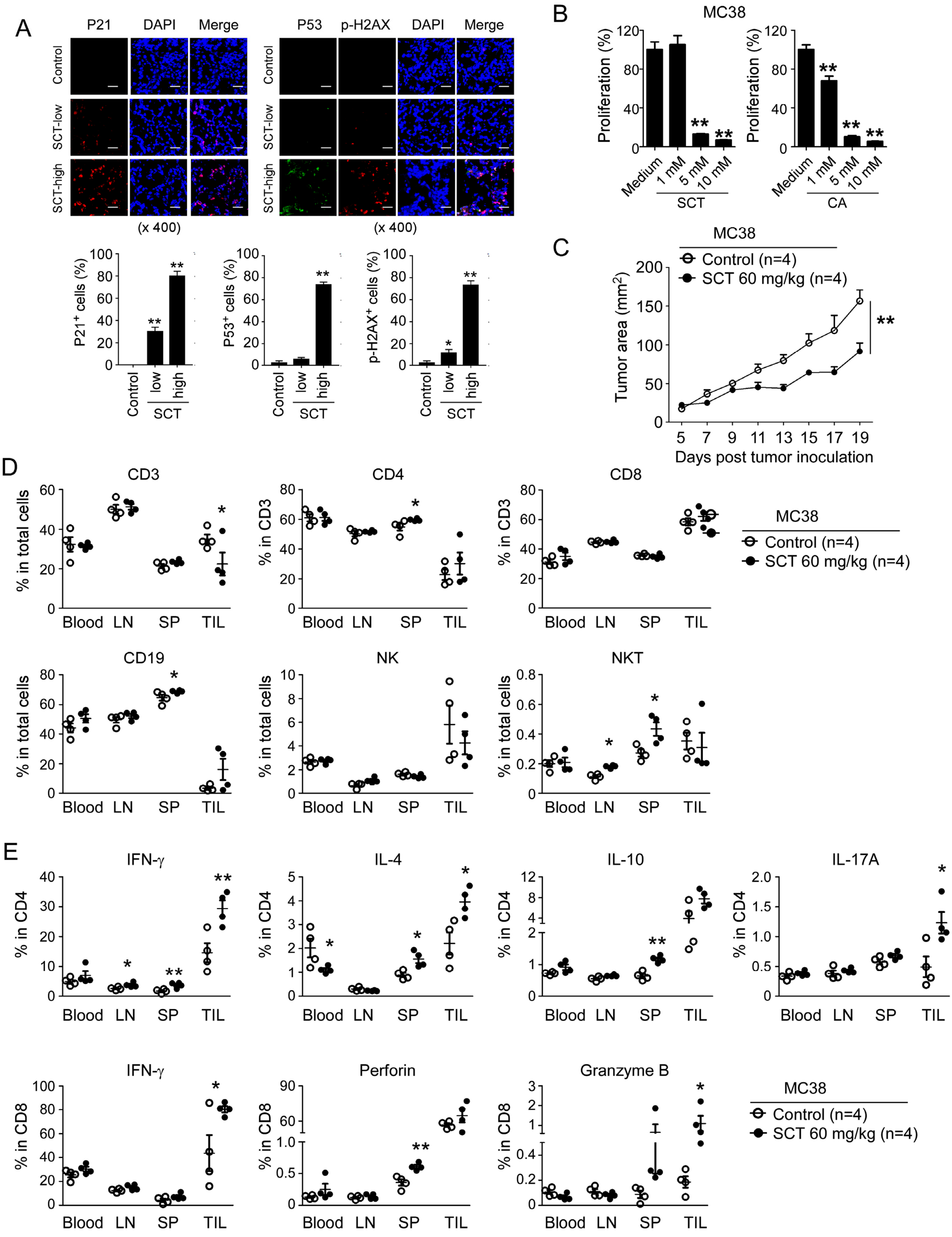
A
P21
DAPI
Merge
Control
SCT-low
SCT-high
(x 400)
P53
p-H2AX
DAPI
Merge
Control
SCT-low
SCT-high
(x 400)
P21+ cells (%)
Control
low
high
SCT
P53+ cells (%)
Control
low
high
SCT
p-H2AX+ cells (%)
Control
low
high
SCT
B
MC38
Proliferation (%)
Medium
1 mM
5 mM
10 mM
SCT
Proliferation (%)
Medium
1 mM
5 mM
10 mM
CA
C
MC38
Control (n=4)
SCT 60 mg/kg (n=4)
Tumor area (mm2)
Days post tumor inoculation
D
CD3
% in total cells
CD4
% in CD3
CD8
% in CD3
MC38
Control (n=4)
SCT 60 mg/kg (n=4)
CD19
% in total cells
NK
% in total cells
NKT
% in total cells
Blood
LN
SP
TIL
E
IFN-γ
% in CD4
IL-4
% in CD4
IL-10
% in CD4
IL-17A
% in CD4
IFN-γ
% in CD8
Perforin
% in CD8
Granzyme B
% in CD8
MC38
Control (n=4)
SCT 60 mg/kg (n=4)

Figure S9. Sodium citrate inhibits tumor growth and enhances anti-tumor immunity in vivo in a syngeneic mouse colon cancer model.

(A) SCT treatment significantly increased $P21^+$, $P53^+$, p-$H2AX^+$ cell populations in the tumor tissues using an immunofluorescence assay. Upper panels are representative images of the molecule expression in tumor tissues from different groups. Lower panels are the summary of mean ± SD of positive cell fractions per high microscope field (× 400) in the tumor tissues from 9 mice of each group. **p<0.001, compared with the PBS control treatment mice. Scale bar: 30 μm. **(B)** Mouse colon cancer MC38 cells were treated with indicated doses of SCT or CA for 72 hours and cell proliferation was measured by the MTT assay. The values of cell proliferation are shown as mean ± SD of three independent experiments. Proliferation of tumor cells with medium only served as 100%. *p<0.05 and **p<0.01, compared with the medium only group. **(C)** SCT Treatment dramatically inhibited MC38 tumor growth in tumor-bearing C57BL/6 mice. MC38 cells (0.5 × 10^6/mouse) were subcutaneously injected into C57BL/6 mice. After 5 days of tumor injection, solvent control or SCT (60 mg/kg body weight) were intraperitoneally injected at every other day for twice. From day 9, SCT (60 mg/kg body weight) were intratumorally injected every day to the end of the experiment. Tumor volumes were measured and presented as mean ± SD (n = 4 mice/group). **p<0.01, compared with the solvent control injection group. **(D)** and **(E)** Immune cell populations were isolated from blood, spleen (SP) and tumors from MC38 tumor-bearing mice and evaluated by flow cytometry for immune cell frequency (in D), as well as IFN-γ^+, granzyme B^+, $perforin^+$ and, IL-4, IL-10 and IL-17A-proucing cell populations (in E). Results shown are mean ± SD (n = 4 mice/group). *p<0.05 and **p<0.01, compared with the solvent control injection group. ANOVA was performed in (A) and (B). Unpaired Student's *t*-test was performed in (C)-(E).

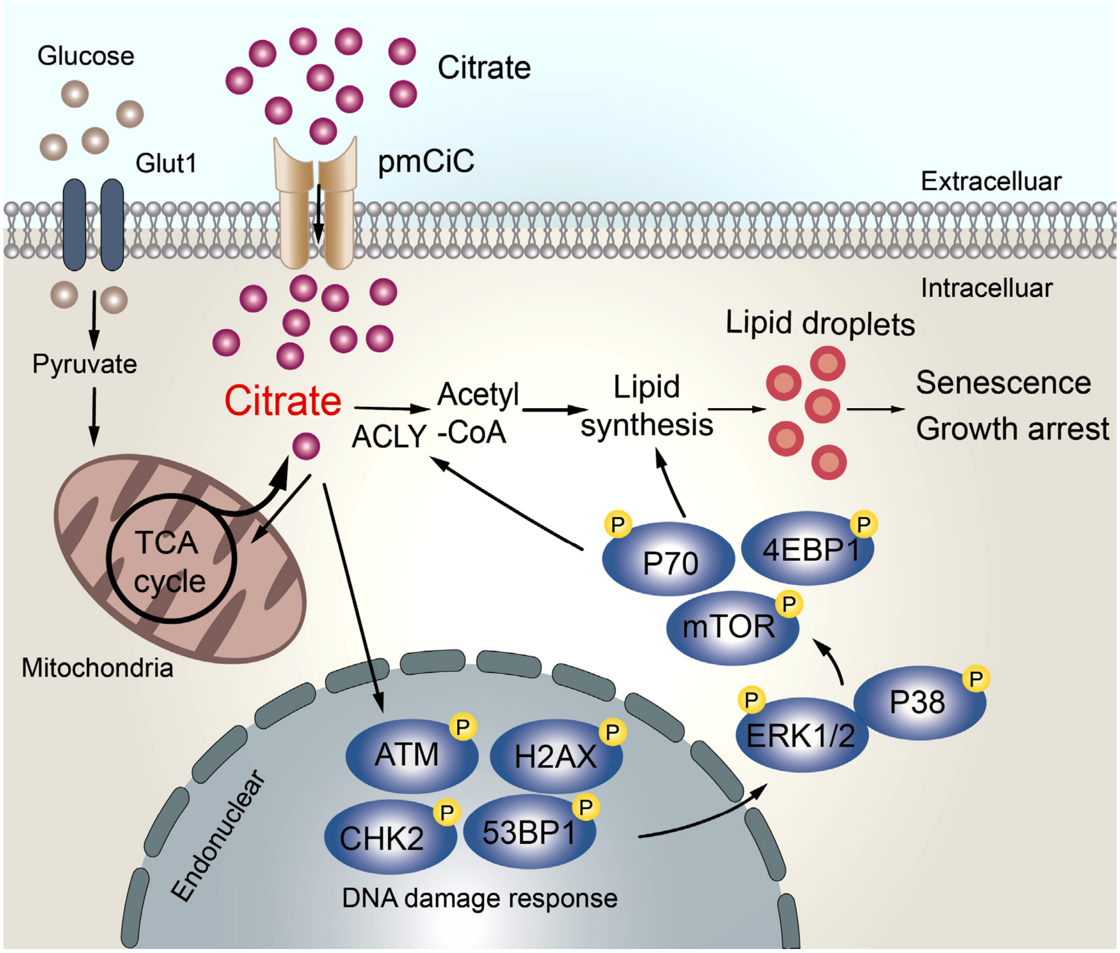

Figure S10. Scheme of extracellular citrate-induced lipid metabolic disturbance and cellular senescence in tumor cells.

In physiological conditions, citrate is synthesized in the TCA cycle from extracellular glucose in mitochondria or transported into the cytoplasm by the transporter pmCiC as a major substrate for lipid synthesis. Extracellular citrate administration in tumor cells induces the phosphorylation of key molecules in the DNA damage response, MAPK and mTOR signaling pathways. The activated cascades induce excessive lipid biosynthesis and provoke senescence to impair tumor cell growth.

Abbreviations: TCA, tricarboxylic acid; pmCiC, plasma membrane citrate transporter; Glut1, glucose transporter 1; ACLY, ATP citrate lyase; MAPK, mitogen-activated protein kinase; mTOR, mammalian target of rapamycin; ATM, ataxia telangiectasia mutated; CHK2, checkpoint kinase 2; 53BP1, p53-binding protein 1; ERK, extracellular signal-regulated kinase.

Supplementary Table 1. Primers used for real-time PCR

Genes	Primers	
	**Forward**	**Reverse**
Glut1	ATTGGCTCCGGTATCGTCAAC	GCTCAGATAGGACATCCAGGGTA
Glut3	GCTCTCTGGGATCAATGCTGTGT	CTTCCTGCCCTTTCCACCAGA
HK2	AACAGCCTGGACGAGAGCAT	GCCAACAATGAGGCCAACTT
GPI	GATGGTAGCTCTCTGCAGCC	GCCATGGCGGGACTCTTG
PFK1	GGCAGCCATGCATAAAGACG	AAGCTTCCCCAGCTGTTCTC
TPI1	AGGCATGTCTTTGGGGAGTC	AGTCCTTCACGTTATCTGCGA
ENO1	CGCCTTAGCTAGGCAGGAAG	GGTGAACTTCTAGCCACTGGG
PKM2	ACGAGAACATCCTGTGGCTG	AGGAAGTCGGCACCTTTCTG
LDHα	AGCTGTTCCACTTAAGGCCC	TGGAACCAAAAGGAATCGGGA
HIF1α	GAACGTCGAAAAGAAAAGTCTCG	CCTTATCAAGATGCGAACTCACA
CPT1	ATCAATCGGACTCTGGAAACGG	TCAGGGAGTAGCGCATGGT
ACC1	TCACACCTGAAGACCTTAAAGCC	AGCCCACACTGCTTGTACTG
FASN	ACAGCGGGGAATGGGTACT	GACTGGTACAACGAGCGGAT
HMGCS1	GTTGGCGGCTATAAAGCTGGT	CCTTCGGGCACAAGCG
HMGCR	GTGAGATCTGGAGGATCCAAGG	GATGGGAGGCCACAAAGAGG
IDI1	CGGAGGCTGATCAGTGTTCTA	TGTTGCTTGTCGAGGTGGTT
SQLE	TGACAATTCTCATCTGAGGTCCA	TCCCAAAAGAAGAACACCTCGT
ACAT1	TACCAGAAGTAAAGCAGCATGG	TCATTCAGTGTACTGGCATTGG
ACAT2	GCGGACCATCATAGGTTCCTT	ACTGGCTTGTCTAACAGGATTCT
cPLA2a	AATACTGCACAATGCCCTTTACC	GCTTCCAAATAAGTCGGGAGC
LIPA	CCCACGTTTGCACTCATGTC	CCCAGTCAAAGGCTTGAAACTT
ACLY	GAAGGGAGTGACCATCATCG	TTAAAGCACCCAGGCTTGAT
CS	GATTGTGCCCAATGTCCTCT	TTCATCTCCGTCATGCCATA
SLC25A1	CCCCATGGAGACCATCAAG	CCTGGTACGTCCCCTTCAG
IL-1β	ACAGATGAAGTGCTCCTTCCA	GTCGGAGATTCGTAGCTGGAT
IL-6	GTAGCCGCCCCACACAGA	CATGTCTCCTTTCTCAGGGCT
IL-8	ATAAAGACATACTCCAAACCTTTCCAC	AAGCTTTACAATAATTTCTGTGTTGGC
TNF	GGAGAAGGGTGACCGACTCA	CTGCCCAGACTCGGCAA
β-actin	TGGCACCCAGCACAATGAA	CTAAGTCATAGTCCGCCTAGAAGCA
